# Supplementary material for: Mimicking on-water surface synthesis through micellar interfaces
Source: Nat Commun. 2024 Dec 3;15:10495. doi: 10.1038/s41467-024-54962-z (PMC11615243; doi:10.1038/s41467-024-54962-z)
Supplement: Supplementary file 1 — Supplementary Information [file 41467_2024_54962_MOESM1_ESM.pdf]

Supplementary Information

Mimicking On-Water Surface Synthesis through Micellar Interfaces

Anupam Prasoon[1,2], Shaik Ghouse[1], Nguyen Ngan Nguyen[1,2], Hyejung Yang[1], Alina Müller[1], Naisa Chandrasekhar[1,2], Silvia Paasch[1], Abdallh Herbawe[3], Muhannad Al Aiti[3], Gianaurelio Cuniberti[3], Eike Brunner[1], Xinliang Feng[1,2]*

[1]Center for Advancing Electronics Dresden (cfaed) and Faculty of Chemistry and Food Chemistry, Technische Universität Dresden, 01062 Dresden, Germany

[2]Max Planck Institute for Microstructure Physics, Halle (Saale) D-06120, Germany

[3]Institute for Materials Science and Max Bergmann Center of Biomaterials, Technische Universität Dresden, 01062 Dresden, Germany

[*]E-mail: xinliang.feng@tu-dresden.de

Table of Contents

Supplementary Methods....4-11

Supplementary Figure 1....12

Supplementary Figure 2....13

Supplementary Figure 3....14

Supplementary Figure 4....15

Supplementary Figure 5 and Supplementary Note 1....16

Supplementary Figure 6 and Supplementary Note 2....17

Supplementary Figure 7 and Supplementary Note 3....18-19

Supplementary Figure 8....19

Supplementary Figure 9....20

Supplementary Figure 10....21

Supplementary Figure 11....22

Supplementary Figure 12 and Supplementary Note 4....23

Supplementary Figure 13 and Supplementary Note 5....24-25

Supplementary Figure 14....26

Supplementary Figure 15....27

Supplementary Figure 16....28

Supplementary Figure 17....29

Supplementary Figure 18....30

Supplementary Figure 19....31

Supplementary Figure 20....32

Supplementary Figure 21....33

Supplementary Figure 22....34

Supplementary Figure 23....35

Supplementary Figure 24....36

Supplementary Figure 25..37

Supplementary Figure 26..38

Supplementary Figure 27..39

Supplementary Figure 28..40

Supplementary Figure 29..41

Supplementary Figure 30..42

Supplementary Figure 31..43

Supplementary Figure 32..44

Supplementary Figure 33..45

Supplementary Figure 34..46

Supplementary Figure 35 and Supplementary Note 6...47

Supplementary Figure 36..48

Supplementary Figure 37..48

Supplementary Figure 38..49

Supplementary Figure 39..50

Supplementary Figure 40..51

Supplementary Figure 41 and Supplementary Note 7...52

Supplementary Figure 42 and Supplementary Note 8...53

Supplementary Figure 43 and Supplementary Note 9...54

Supplementary Figure 44 and Supplementary Note 10...55

Supplementary References...56

Supplementary Methods

The chemicals 5-(4-aminophenyl)-10,15,20-(triphenyl)porphyrin (R1) and 5-(4-carboxyphenyl)-10,15,20-(triphenyl)porphyrin (R3), were obtained from PorphyChem. The monomers M1, M2, M3, and M4 used in our study were commercially sourced. Specifically, M1 and M3 were purchased from PorphyChem, M2 was obtained from TCI, and M4 was acquired from Sigma-Aldrich. All monomers were used directly without further purification. .Other chemicals (R2, R4 - R15) and solvents, such as chloroform and THF, were obtained from PorphyChem, abcr GmbH, and Sigma-Aldrich and were used without further purification. Purified water was obtained through a Milli-Q purification system (Merck KGaA). All site-selective chemical reactions were carried out under ambient atmospheric conditions and at room temperature. The substrates used, including 300 nm SiO_2/Si wafers, quartz glass, and copper grids for TEM, were obtained from Microchemicals and Plano GmbH.

Site-selective reaction SSR-1 on an anionic micelle surface

Micelles of sodium oleyl sulfate (SOS), an anionic surfactant, were prepared by dissolving 20 mg of SOS (2.87 mmol/L) in an aqueous solution above its critical micelle concentration (CMC) of 1.7 mmol/L. This solution was placed in a 100 mL Duran glass bottle with a GL-45 cap. Then, R1 (1.59 µmol), dissolved in 20 mL of 0.15 M HCl aqueous solution, was injected into the bottle. After 45 minutes, R2 (7.94 µmol), dissolved in 20 mL of 0.05 M LiOH aqueous solution, was added to the bottle. After 10 hours, the precipitate was washed sequentially with water and THF, and then dried under high vacuum. This resulted in the isolation of the pure site-selective product SSC-1, 1.3 mg, with a 96% yield. ^{1}H NMR (300 MHz, DMSO-d_6, 30 °C) δ = 8.97 (d, J = 4.7 Hz, 2H), 8.80 (d, J = 4.7 Hz, 5H), 8.39 (s, 2H), 8.23 (ddd, J = 7.5, 4.8, 2.4 Hz, 7H), 8.01 (s, 2H), 7.97 – 7.75 (m, 11H), 7.03 (d, J = 8.4 Hz, 2H), -3.01 (s, 2H). Mass spectra +m/z had been calculated [$C_{58}H_{33}N_5O_5$] = 879.25; found 879.21.

Site-selective reaction SSR-2 on an anionic micelle surface

Micelles of sodium oleyl sulfate (SOS), an anionic surfactant, were prepared by dissolving 20 mg of SOS (2.87 mmol/L) in an aqueous solution above its critical micelle concentration (CMC) of 1.7 mmol/L. This solution was placed in a 100 mL Duran glass bottle with a GL-45 cap. Then, R1 (1.59 µmol), dissolved in 20 mL of 0.15 M HCl aqueous solution, was injected into the bottle. After 45 minutes, R4 (7.94 µmol), dissolved in 20 mL of 0.05 M LiOH aqueous solution, was added to the bottle. After 10 hours, the precipitate was washed sequentially with

water and THF, and then dried under high vacuum. This resulted in the isolation of the pure site-selective insoluble product SSC-2, 1.6 mg, with a 98% yield. Mass spectra +m/z had been calculated [$C_{68}H_{37}N_5O_5$] = 1003.28; found 1003.23.

Site-selective reaction SSR-3 on an anionic micelle surface

Micelles of sodium oleyl sulfate (SOS), an anionic surfactant, were prepared by dissolving 20 mg of SOS (2.87 mmol/L) in an aqueous solution above its critical micelle concentration (CMC) of 1.7 mmol/L. This solution was placed in a 100 mL Duran glass bottle with a GL-45 cap. Then, R1 (1.59 µmol), dissolved in 20 mL of 0.15 M HCl aqueous solution, was injected into the bottle. After 45 minutes, R9 (7.94 µmol), dissolved in 20 mL of 0.05 M LiOH aqueous solution, was added to the bottle. After 10 hours, the precipitate was washed sequentially with water and THF, and then dried under high vacuum. This resulted in the isolation of the pure site-selective product SSC-3, 1.2 mg, with a 98% yield. ^{1}H NMR (300 MHz, DMSO-d_6, 30 °C) δ = 10.08 (s, 1H), 8.77-8.39 (m, 17H), 8.1-7.96 (m, 12H), 7.90-7.62 (m, 4H). Mass spectra +m/z had been calculated [$C_{50}H_{33}N_5OS$] = 751.24; found 751.22.

Site-selective reaction SSR-4 on an anionic micelle surface

Micelles of sodium oleyl sulfate (SOS), an anionic surfactant, were prepared by dissolving 20 mg of SOS (2.87 mmol/L) in an aqueous solution above its critical micelle concentration (CMC) of 1.7 mmol/L. This solution was placed in a 100 mL Duran glass bottle with a GL-45 cap. Then, R1 (1.59 µmol), dissolved in 20 mL of 0.15 M HCl aqueous solution, was injected into the bottle. After 45 minutes, R12 (7.94 µmol), dissolved in 20 mL of 0.05 M LiOH aqueous solution, was added to the bottle. After 10 hours, the precipitate was washed sequentially with water and THF, and then dried under high vacuum. This resulted in the isolation of the pure site-selective insoluble product SSC-4, 1.3 mg, with a 97% yield. Mass spectra +m/z had been calculated [$C_{54}H_{35}N_5OS_2$] = 833.23; found 833.21.

Site-selective reaction SSR-5 on an anionic micelle surface

Micelles of sodium oleyl sulfate (SOS), an anionic surfactant, were prepared by dissolving 20 mg of SOS (2.87 mmol/L) in an aqueous solution above its critical micelle concentration (CMC) of 1.7 mmol/L. This solution was placed in a 100 mL Duran glass bottle with a GL-45 cap. Then, R1 (1.59 µmol), dissolved in 20 mL of 0.15 M HCl aqueous solution, was injected

into the bottle. After 45 minutes, R7 (7.94 μmol), dissolved in 20 mL of 0.05 M LiOH aqueous solution, was added to the bottle. After 10 hours, the precipitate was washed sequentially with water and THF, and then dried under high vacuum. This resulted in the isolation of the pure site-selective product SSC-5, 1.1 mg, with a 98% yield. ^{1}H NMR (300 MHz, DMSO-d_6, 30 °C) δ = 10.14 (s, 1H), 8.92-8.48 (m, 10H), 8.32-7.99 (m, 15H), 7.89- 7.77 (m, 6H). Mass spectra +m/z had been calculated [$C_{52}H_{35}N_5O$] = 745.28; found 745.26.

Site-selective reaction SSR-6 on an anionic micelle surface

Micelles of sodium oleyl sulfate (SOS), an anionic surfactant, were prepared by dissolving 20 mg of SOS (2.87 mmol/L) in an aqueous solution above its critical micelle concentration (CMC) of 1.7 mmol/L. This solution was placed in a 100 mL Duran glass bottle with a GL-45 cap. Then, R1 (1.59 μmol), dissolved in 20 mL of 0.15 M HCl aqueous solution, was injected into the bottle. After 45 minutes, R8 (7.94 μmol), dissolved in 20 mL of 0.05 M LiOH aqueous solution, was added to the bottle. After 10 hours, the precipitate was washed sequentially with water and THF, and then dried under high vacuum. This resulted in the isolation of pure site-selective product SSC-6, 1.3 mg with a 98% yield. ^{1}H NMR (300 MHz, DMSO-d_6, 30 °C) δ = 10.09 (s, 1H), 8.84 (s, 2H), 8.74-8.40 (m, 7H), 8.30- 7.97 (m, 22H), 7.94-7.29 (m, 5H). Mass spectra +m/z had been calculated [$C_{58}H_{41}N_5O$] = 821.31; found 821.28.

Site-selective reaction SSR-7 on an anionic micelle surface

Micelles of sodium oleyl sulfate (SOS), an anionic surfactant, were prepared by dissolving 20 mg of SOS (2.87 mmol/L) in an aqueous solution above its critical micelle concentration (CMC) of 1.7 mmol/L. This solution was placed in a 100 mL Duran glass bottle with a GL-45 cap. Then, R1 (1.59 μmol), dissolved in 20 mL of 0.15 M HCl aqueous solution, was injected into the bottle. After 45 minutes, R5 (7.94 μmol), dissolved in 20 mL of 0.05 M LiOH aqueous solution, was added to the bottle. After 10 hours, the precipitate was washed sequentially with water and THF, and then dried under high vacuum. This resulted in the isolation of pure site-selective product SSC-7, 1.2 mg with a 98% yield. ^{1}H NMR (300 MHz, DMSO-d_6, 30 °C) δ = 10.3 (s, 1H), 9.00-8.77 (m, 4H), 8.76-8.38 (m, 8H), 8.36- 8.00 (m, 9H), 7.93-7.77 (m, 5H), 7.74-7.20 (m, 4H). Mass spectra +m/z had been calculated [$C_{52}H_{37}N_5O3$] = 777.27; found 777.22.

Site-selective reaction SSR-8 on an anionic micelle surface

Micelles of sodium oleyl sulfate (SOS), an anionic surfactant, were prepared by dissolving 20 mg of SOS (2.87 mmol/L) in an aqueous solution above its critical micelle concentration (CMC) of 1.7 mmol/L. This solution was placed in a 100 mL Duran glass bottle with a GL-45 cap. Then, R1 (1.59 µmol), dissolved in 20 mL of 0.15 M HCl aqueous solution, was injected into the bottle. After 45 minutes, R10 (7.94 µmol), dissolved in 20 mL of 0.05 M LiOH aqueous solution, was added to the bottle. After 10 hours, the precipitate was washed sequentially with water and THF, and then dried under high vacuum. This resulted in the isolation of pure site-selective product SSC-8, 1.2 mg with a 92% yield. ^{1}H NMR (300 MHz, DMSO-d_6, 30 °C) δ = 8.84 (s, 4H), 8.76-8.38 (m, 10H), 8.31-7.77 (m, 16H). Mass spectra +m/z had been calculated [$C_{54}H_{33}N_5O_6$] = 847.24; found 847.20.

Site-selective reaction SSR-9 on an anionic micelle surface

Micelles of sodium oleyl sulfate (SOS), an anionic surfactant, were prepared by dissolving 20 mg of SOS (2.87 mmol/L) in an aqueous solution above its critical micelle concentration (CMC) of 1.7 mmol/L. This solution was placed in a 100 mL Duran glass bottle with a GL-45 cap. Then, R1 (1.59 µmol), dissolved in 20 mL of 0.15 M HCl aqueous solution, was injected into the bottle. After 45 minutes, R11 (7.94 µmol), dissolved in 20 mL of 0.05 M LiOH aqueous solution, was added to the bottle. After 10 hours, the precipitate was washed sequentially with water and THF, and then dried under high vacuum. This resulted in the isolation of pure site-selective product SSC-9, 1.1 mg with a 95% yield. Mass spectra +m/z had been calculated [$C_{52}H_{35}N_5O_3$] = 777.27; found 777.22.

Site-selective reaction SSR-10 on cationic micelle surface

Micelles of cetyltrimethylammonium bromide (CTAB), a cationic surfactant, were prepared by dissolving 30 mg of CTAB (4.11 mmol/L) in an aqueous solution above its critical micelle concentration (CMC) of 1.1 mmol/L. This solution was placed in a 100 mL Duran glass bottle with a GL-45 cap. Then, R3 (1.52 µmol), dissolved in 20 mL of 0.08 M LiOH aqueous solution, was injected into the bottle. After 45 minutes, R6 (7.59 µmol), dissolved in 20 mL of 0.12 M HCl aqueous solution, was added to the bottle. After 10 hours, the precipitate was washed sequentially with water and THF, and then dried under high vacuum. This resulted in the isolation of pure site-selective product SSC-10, 1.1 mg, with a 98% yield. ^{1}H NMR (300 MHz, DMSO-d_6, 30 °C) δ = 8.85 (s, 8H), 8.37 (q, J = 8.5 Hz, 4H), 8.23 (dd, J = 7.2, 2.1 Hz, 6H), 7.92

– 7.78 (m, 9H), -2.92 (s, 2H). Mass spectra +m/z had been calculated $[C_{51}H_{37}N_8]^+$ = M+H; 861.31; found 861.28.

Site-selective reaction SSR-11 on cationic micelle surface

Micelles of cetyltrimethylammonium bromide (CTAB), a cationic surfactant, were prepared by dissolving 30 mg of CTAB (4.11 mmol/L) in an aqueous solution above its critical micelle concentration (CMC) of 1.1 mmol/L. This solution was placed in a 100 mL Duran glass bottle with a GL-45 cap. Then, R3 (1.52 μmol), dissolved in 20 mL of 0.08 M LiOH aqueous solution, was injected into the bottle. After 45 minutes, R13 (7.59 μmol), dissolved in 20 mL of 0.12 M HCl aqueous solution, was added to the bottle. After 10 hours, the precipitate was washed sequentially with water and THF, and then dried under high vacuum. This resulted in the isolation of pure site-selective product SSC-11, 1.1 mg with a 97% yield. Mass spectra +m/z had been calculated $[C_{51}H_{34}N_6S_2]$ = M; 794.23; found 794.20.

Site-selective reaction SSR-12 on cationic micelle surface

Micelles of cetyltrimethylammonium bromide (CTAB), a cationic surfactant, were prepared by dissolving 30 mg of CTAB (4.11 mmol/L) in an aqueous solution above its critical micelle concentration (CMC) of 1.1 mmol/L. This solution was placed in a 100 mL Duran glass bottle with a GL-45 cap. Then, R3 (1.52 μmol), dissolved in 20 mL of 0.08 M LiOH aqueous solution, was injected into the bottle. After 45 minutes, R14 (7.59 μmol), dissolved in 20 mL of 0.12 M HCl aqueous solution, was added to the bottle. After 10 hours, the precipitate was washed sequentially with water and THF, and then dried under high vacuum. This resulted in the isolation of pure site-selective product SSC-12, 1.1 mg with a 97% yield. ^{1}H NMR (300 MHz, DMSO-d_6, 30 °C) δ = 8.83 (s, 1H), 8.7-8.6 (m, 2H), 8.39-7.98 (m, 26H), 7.87 – 7.72 (m, 1H). Mass spectra +m/z had been calculated $[C_{51}H_{34}N_6O_2]$ = M; 762.27; found 762.28.

Site-selective reaction SSR-13 on an anionic micelle surface

Micelles of sodium oleyl sulfate (SOS), an anionic surfactant, were prepared by dissolving 20 mg of SOS (2.87 mmol/L) in an aqueous solution above its critical micelle concentration (CMC) of 1.7 mmol/L. This solution was placed in a 100 mL Duran glass bottle with a GL-45 cap. Then, R1 (1.59 μmol), dissolved in 20 mL of 0.15 M HCl aqueous solution, was injected into the bottle. After 45 minutes, R15 (7.94 μmol), dissolved in 20 mL of 0.05 M LiOH aqueous solution, was added to the bottle. After 10 hours, the precipitate was washed sequentially with

water and THF, and then dried under high vacuum. This resulted in the isolation of pure site-selective product SSC-13, 1.6 mg with a 96% yield. ^{1}H NMR (300 MHz, DMSO-d_6, 30 °C) δ = 8.90-8.73 (m, 2H), 8.74-8.40 (m, 19H), 8.39-8.31 (d, 2H), 8.27 – 7.95 (m, 15H), 7.93-7.75 (m, 5H), 7.70-7.21 (m, 8H). Mass spectra +m/z had been calculated [$C_{78}H_{53}N_5O^{2+}$] = M; 1075.42; found 1075.41.

Site-selective reaction SSR-14 on cationic micelle surface

Micelles of cetyltrimethylammonium bromide (CTAB), a cationic surfactant, were prepared by dissolving 30 mg of CTAB (4.11 mmol/L) in an aqueous solution above its critical micelle concentration (CMC) of 1.1 mmol/L. This solution was placed in a 100 mL Duran glass bottle with a GL-45 cap. Then, R3 (1.52 µmol), dissolved in 20 mL of 0.08 M LiOH aqueous solution, was injected into the bottle. After 45 minutes, R11 (7.94 µmol), dissolved in 20 mL of 0.12 M HCl aqueous solution, was added to the bottle. After 10 hours, the precipitate was washed sequentially with water and THF, and then dried under high vacuum. This resulted in the isolation of pure site-selective product SSC-14, 1.1 mg with a 93% yield. ^{1}H NMR (300 MHz, DMSO-d_6, 30 °C) δ = 8.85 (s, 8H), 8.37 (q, J = 8.3 Hz, 4H), 8.24 (dd, J = 7.1, 2.2 Hz, 6H), 8.02 (s, 4H), 7.84 (dd, J = 8.6, 3.2 Hz, 9H), 2.91 (s, 2H). Mass spectra +m/z had been calculated [$C_{53}H_{34}N_4O_5$] = 806.25; found 806.21.

Site-Selective Reaction SSR-1 on an anionic micelle surface (Scaled up reaction)

Micelles of sodium oleyl sulfate (SOS), an anionic surfactant, were prepared by dissolving 50 mg of SOS (2.87 mmol/L) in an aqueous solution above its critical micelle concentration (CMC) of 1.7 mmol/L. This solution was placed in a 250 mL Duran glass bottle with a GL-45 cap. Then, R1 (7.94 µmol), dissolved in 100 mL of 0.15 M HCl aqueous solution, was injected into the bottle. After 45 minutes, R2 (15.88 µmol), dissolved in 40 mL of 0.05 M LiOH aqueous solution, was added to the bottle. After 10 hours, the precipitate was washed sequentially with water and THF, and then dried under high vacuum. This resulted in the isolation of pure site-selective product SSC-1, 6.5 mg, with a 93% yield. ^{1}H NMR (300 MHz, DMSO-d_6, 30 °C) δ = 8.97 (d, J = 4.7 Hz, 2H), 8.80 (d, J = 4.7 Hz, 5H), 8.39 (s, 2H), 8.23 (ddd, J = 7.5, 4.8, 2.4 Hz, 7H), 8.01 (s, 2H), 7.97 – 7.75 (m, 11H), 7.03 (d, J = 8.4 Hz, 2H), -3.01 (s, 2H). Mass spectra +m/z had been calculated [$C_{58}H_{33}N_5O_5$] = 879.25; found 879.21.

Site-selective reaction SSR-10 on cationic micelle surface (Scaled up reaction)

Micelles of cetyltrimethylammonium bromide (CTAB), a cationic surfactant, were prepared by dissolving 50 mg of CTAB (4.11 mmol/L) in an aqueous solution above its critical micelle concentration (CMC) of 1.1 mmol/L. This solution was placed in a 250 mL Duran glass bottle with a GL-45 cap. Then, R3 (7.59 µmol), dissolved in 100 mL of 0.08 M LiOH aqueous solution, was injected into the bottle. After 45 minutes, R6 (15.18 µmol), dissolved in 40 mL of 0.12 M HCl aqueous solution, was added to the bottle. After 10 hours, the precipitate was washed sequentially with water and THF, and then dried under high vacuum. This resulted in the isolation of pure site-selective product SSC-10, 5.4 mg, with a 94% yield. ^{1}H NMR (300 MHz, DMSO-d$_6$, 30 °C) δ = 8.85 (s, 8H), 8.37 (q, J = 8.5 Hz, 4H), 8.23 (dd, J = 7.2, 2.1 Hz, 6H), 7.92 – 7.78 (m, 9H), -2.92 (s, 2H). Mass spectra +m/z had been calculated $[C_{51}H_{37}N_8]^+$ = M+H; 861.31; found 861.28.

Synthesis of 2DPI on an anionic micelle surface

Micelles of sodium oleyl sulfate (SOS), an anionic surfactant, were prepared by dissolving 60 mg of SOS (2.87 mmol/L) in an aqueous solution above its critical micelle concentration (CMC) of 1.7 mmol/L. This solution was placed in a 250 mL Duran glass bottle with a GL-45 cap. Then, M1 (29.64 µmol), dissolved in 100 mL of 0.15 M HCl aqueous solution, was injected into the bottle. After 45 minutes, M2 (59.28 µmol), dissolved in 50 mL of 0.05 M LiOH aqueous solution, was added to the bottle. After 18 hours, the precipitate was washed sequentially with water and THF, and then dried under high vacuum. This resulted in the isolation of the pure site-selective product SSC-1, 24.9 mg, with a 92% yield.

Synthesis of 2DPBI on cationic micelle surface

Micelles of cetyltrimethylammonium bromide (CTAB), a cationic surfactant, were prepared by dissolving 60 mg of CTAB (4.11 mmol/L) in an aqueous solution above its critical micelle concentration (CMC) of 1.1 mmol/L. This solution was placed in a 250 mL Duran glass bottle with a GL-45 cap. Then, M3 (37.94 µmol), dissolved in 100 mL of 0.08 M LiOH aqueous solution, was injected into the bottle. After 45 minutes, M4 (75.87 µmol), dissolved in 50 mL of 0.12 M HCl aqueous solution, was added to the bottle. After 18 hours, the precipitate was washed sequentially with water and THF, and then dried under high vacuum. This resulted in the isolation of the pure site-selective product 2DPBI, 31.2 mg, with a 94% yield.

Synthesis of 2DPI via solvothermal bulk organic synthesis

2,5-dihydroxyterephthalaldehyde (M2) (13.3 mg, 0.08 mmol) and 5,10,15,20-(tetra-4-aminophenyl)porphyrin (M1) (27.0 mg, 0.04 mmol) in presence of 6 M acetic acid (0.2 mL) using dichlorobenzene, ethanol (1:1) as solvent combination (2 mL). This mixture was sonicated for 10-15 minutes in order to get a homogenous dispersion. The tube was then flash frozen at 77 K (liquid N_2 bath) and degassed by three freeze-pump-thaw cycles. The tube was sealed off and then heated at 120°C for 3 days. After the reaction the powders are filtered out, washed with ethanol and dried under vacuum at 150 °C for 12 hours, yielding a purple colored powder in 79 % (28 mg) isolated yield[1].

Synthesis of 2DPBI via solvothermal bulk organic synthesis

A 10mL sealed tube filled with argon was charged with 10 mg (13 μmol) 5,10,15,20-(tetra-4-carboxyphenyl)porphyrin (M3) 7.18 mg (25 μmol) of 1,2,4,5-benzenetetramine tetrahydrochloride (M4) and in the presence of 2 mL of polyphosphoric acid (PPA) as solvent and catalyst under inert atmosphere. The reaction was stirred at 150 °C for 24 h and then stirred at 180 °C for 24 h. After the pH of the resulting mixture was adjusted to 8-9 with saturated sodium hydrogen carbonate ($NaHCO_3$), 2D-polymer was precipitated. After filtration, the polymer was obtained as dark brownish colored powder. The powder collected and then soxhlet extraction was performed with water, methanol, acetone for 12 hours and then dried at 100°C under vacuum for 6 hours, yielding a black colored powder in 77% (11.5 mg), isolated yield[2].

Supplementary Figures

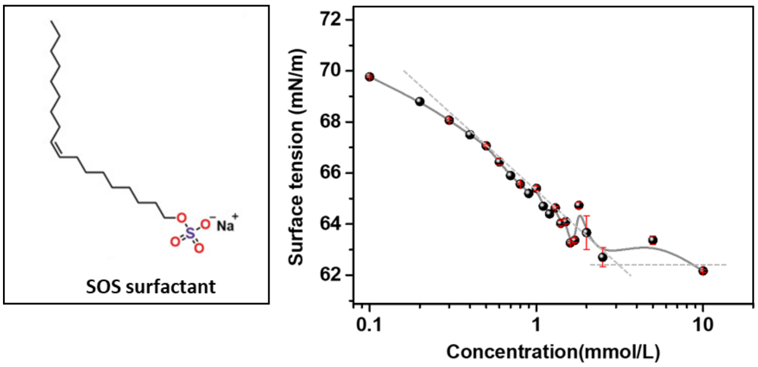

Supplementary Fig. 1. Chemical structure of sodium oleyl sulfate (SOS), an anionic surfactant, illustrating its amphiphilic nature with a hydrophobic alkyl chain and a hydrophilic sulfate group. Surface tension (mN/m) versus logarithm of concentration (mmol/L) plot for SOS in aqueous solution, demonstrating the characteristic behavior of surfactants. The inflection point on the curve indicates the critical micelle concentration (CMC), where a sharp change in surface tension is observed.

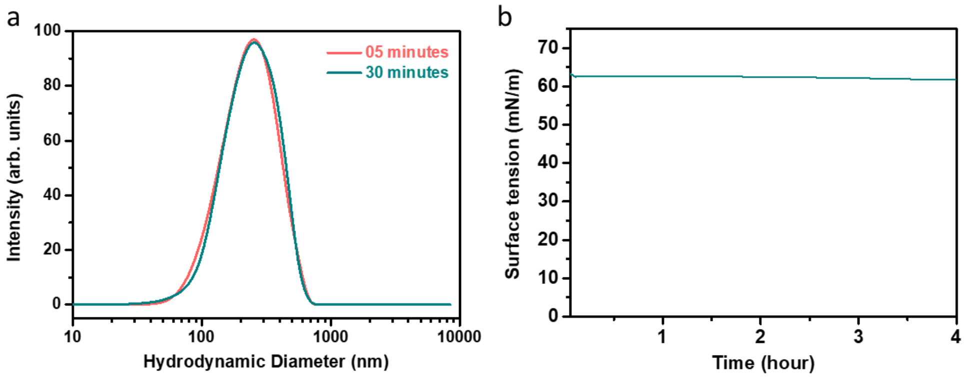

Supplementary Fig. 2. Time-dependent DLS measurements showed that the hydrodynamic diameter of SOS micelles remained constant over 30 minutes, indicating stable micelle structures. Additionally, time-dependent surface tension measurements illustrates the stability of the surface tension over an extended period, highlighting the stability of the SOS micelles in the solution.

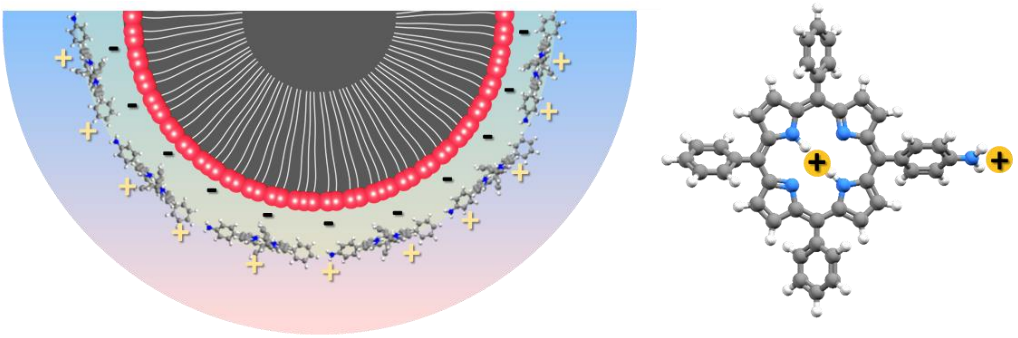

R1

Supplementary Fig. 3. Following the formation of micelles, Step II involves introducing the protonated R1 molecule (at pH ~1.2) into the aqueous solution. The positive charge on the protonated R1 molecules facilitates their electrostatic interaction with the negatively charged sulfate (SO_4^-) head groups of the SOS micelles. This interaction results in the pre-assembly of R1 molecules on the surfaces of the SOS micelles.

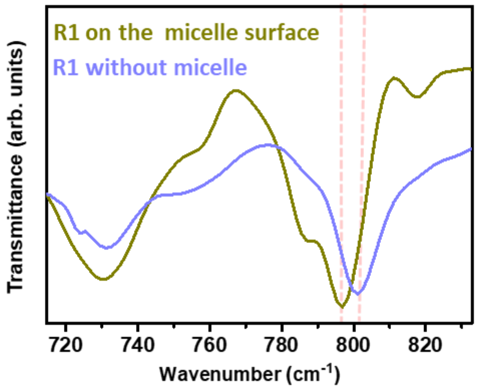

Supplementary Fig. 4. ATR-FTIR spectroscopy analysis of R1 on micelle surfaces and in aqueous bulk. The plot shows a significant red shift in the NH_2-wagging mode from 803 to 795 cm^{-1} on the micelle surface compared to the aqueous bulk. These findings suggest the formation of a J-aggregated structure with a short slip distance in the R1 assembly, indicative of strong polarized-π interactions within the R1 assembled structure on the micelle surface.

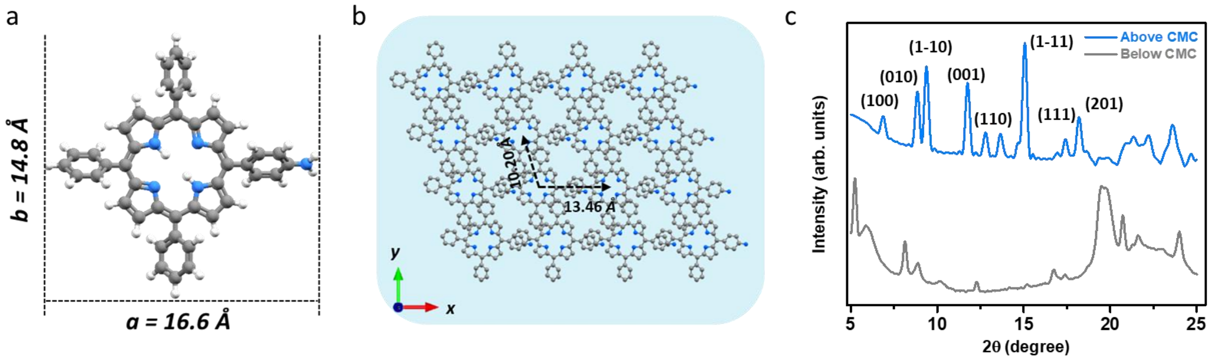

Supplementary Fig. 5. Structural analysis of R1 assembly on micelle surfaces using PXRD. (a) Molecular structure of an individual R1 molecule. (b) Lattice structure of the pre-assembled J-aggregate on the micelle surface. (c) PXRD analysis of R1 molecules above (blue) and below (gray) the CMC of SOS surfactant. Above the CMC, J-aggregation is observed with a smaller unit cell, while below the CMC, a different structure with a larger unit cell and no J-aggregation is present, highlighting the role of micelles in promoting J-aggregation.

Supplementary Note 1

The dimensions of individual R1 porphyrin molecules, measured using Mercury 3.10.1 software, and serve as a baseline for comparison with the J-aggregated structures. The unit cell parameters of the J-aggregates formed on the micelle surface are significantly reduced compared to those of the individual R1 molecules, with observed lattice parameters of approximately 1.34 nm and 1.02 nm. This reduction in lattice size indicates enhanced packing and ordering of the porphyrins within the J-aggregates, characterized by a short slipping distance. Additionally, PXRD experiments to compare the aggregation behavior of R1 molecules above and below the critical micelle concentration (CMC) of the SOS surfactant. Above the CMC, where micelles are present, the PXRD data confirmed the formation of a strongly J-aggregated structure with a smaller unit cell. Conversely, below the CMC, where micelles do not form, the PXRD analysis revealed a distinctly different and less ordered structure with a larger unit cell, indicating the absence of J-aggregation.

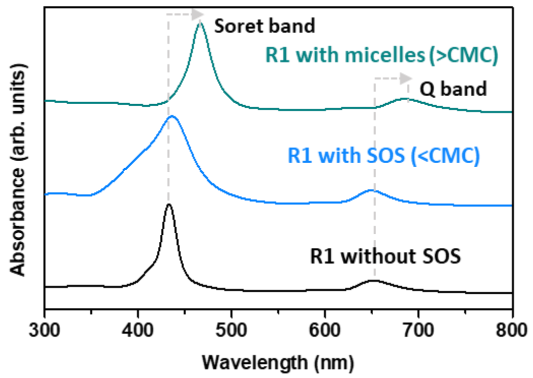

Supplementary Fig. 6. UV-visible absorption spectra of R1 in the presence and absence of SOS surfactant at concentrations above and below the critical micelle concentration (CMC) in water. The R1 spectrum without SOS surfactant serves as a reference.

Supplementary Note 2

We performed UV-visible spectroscopy on R1 both above and below the CMC of SOS surfactant. At concentrations above the CMC, where micelles are present, R1 exhibits a pronounced red shift in the Soret band along with a sharper absorption peak. This red shift is indicative of J-aggregate formation for R1. Conversely, at SOS concentrations below the CMC, the UV-visible absorption spectrum of R1 is significantly broadened, and the characteristic red shift of the Soret band is absent. This broadening suggests that the porphyrin molecules are not forming ordered J-aggregates. Instead, the broad spectrum below the CMC indicates a different, less ordered aggregation or individual porphyrin molecules in solution. This comparison clearly demonstrates that the micelle surface (template) is crucial for the pre-assembly of R1.

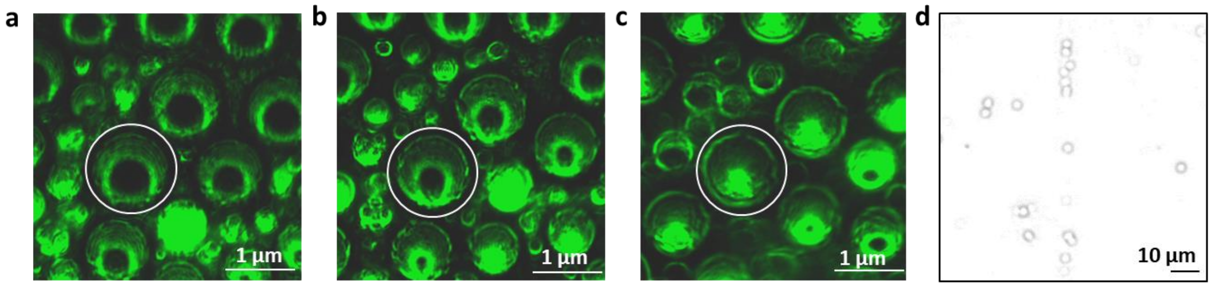

Supplementary Fig. 7. Sequences (a, b, c) of the emitted fluorescence at different focal levels. The black color indicates the hollow core of the micelles and the absence of the SSC-1 product inside. (d) Gray-scale visualization of the emitted fluorescence intensity upon laser excitation, showing no light intensity recorded in the hollow body of the micelles.

Supplementary Note 3

Upon completing Step-III, where the SSC-1 product was formed on the micelle surface, the hollow micelles exhibited bright fluorescence signals along their surface boundaries when viewed under the microscope. By exciting the sample at a specific wavelength (552 nm) corresponding to the excitation spectrum of SSC-1, the emitted light was captured and analyzed. Adjusting the focal plane of the microscope (from panels a to c) allowed us to follow and visualize the fluorescence pattern at the outer edge of the micelles.

Regions of irregular or weak fluorescence, appearing as dark areas, indicate zones not illuminated by the fluorescent laser. For example, in the micelle labeled with a red circle in panels a, b, and c, adjusting the focal plane towards the observer revealed high fluorescence intensity at the edges but not within the central black region, which gradually closed as the focal plane was moved further (panel b). The black area signifies the absence of emitted fluorescence, confirming that the internal core of the micelle is hollow and does not contain any reactant molecules (R1 or R2) within its hydrophobic core. Additionally, gray-scale visualization of the emitted fluorescence intensity upon laser excitation confirmed no light

intensity within the hollow core of the micelles, indicating the absence of SSC-1 product inside the micelles.

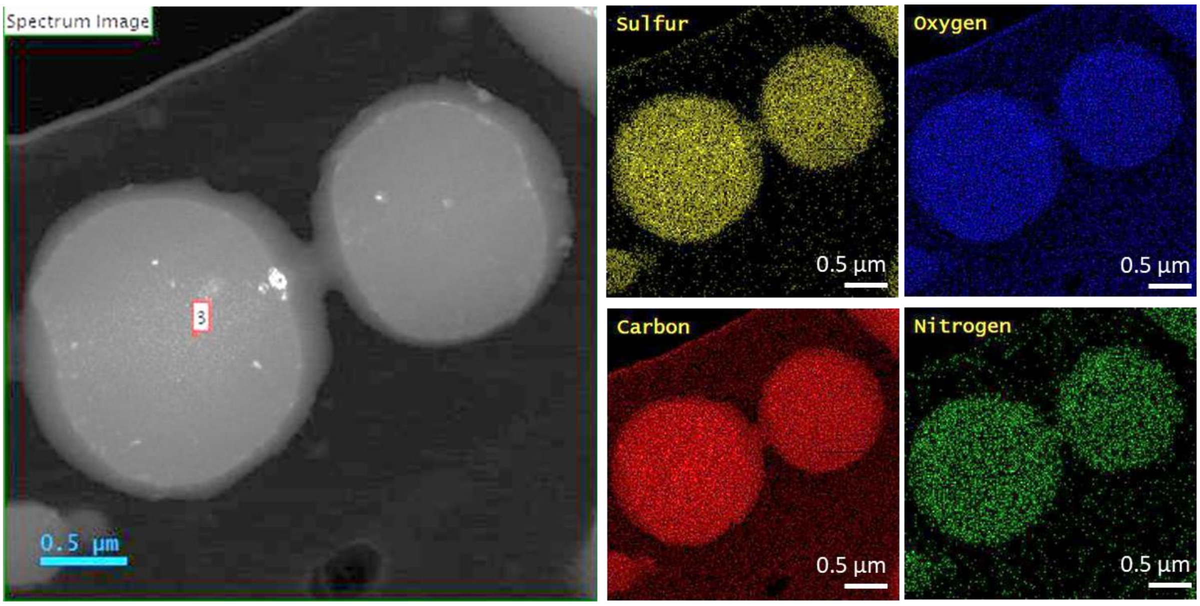

Supplementary Fig. 8. Elemental mapping using Energy Dispersive X-ray Spectroscopy (EDS) in Scanning Transmission Electron Microscopy (STEM) confirmed the presence of characteristic elements from SSC-1 and SOS micelles, supporting the uniform multilayer growth of SSC-1 on the micelle surface.

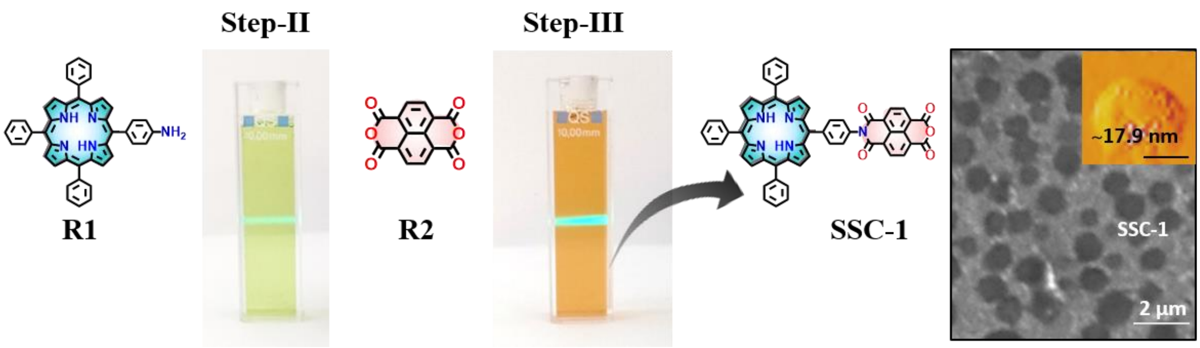

Supplementary Fig. 9. Introduction of naphthalene tetracarboxylic dianhydride (R2) and subsequent changes in micelle characteristics. In Step-III, R2 reactant molecules were added to the aqueous solution containing R1, resulting in a rapid and noticeable color transition from light green to dark orange within a minute and exhibiting a significant Tyndall effect. This color change indicates the efficient chemical reaction between R1 and R2 to form an imide bond. Dynamic light scattering (DLS) analysis showed that the overall size of the micelles increased, reaching approximately 1 μm after 10 hours. Field-emission scanning electron microscopy (FE-SEM) revealed a spherical morphology with an average diameter of ~1 μm and atomic force microscopy (AFM) analysis confirmed the presence of circular sheet structures with a thickness of approximately 18 nm with a scale bar of 0.5 μm.

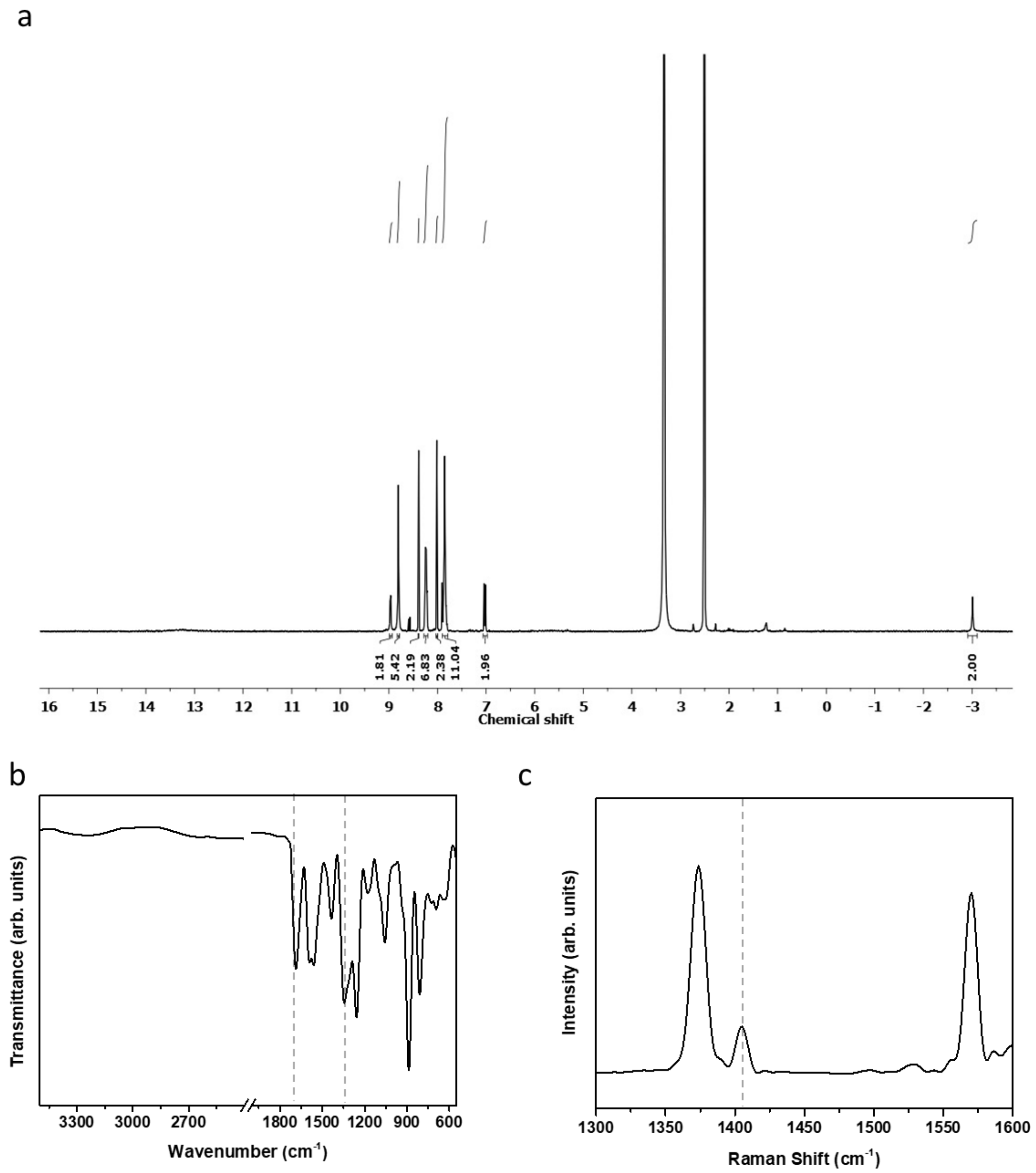

Supplementary Fig. 10. Characterization of site-selective product SSC-1 using ^{1}H NMR, ATR-FTIR, and Raman spectroscopy. (a) The ^{1}H NMR (300 MHz) spectrum of SSC-1 in DMSO-d^6 solvent displays distinct peaks corresponding to the expected chemical shifts. (b) ATR-FTIR spectroscopy of SSC-1 shows the appearance of the imide C–N bond at ~1348 cm^{-1} and the imide C=O bond at ~1684 cm^{-1}, as well as the complete vanishing of the N-H stretch at ~3340 cm^{-1} from R1. (c) The Raman spectra of SSC-1 reveal a new peak at ~1410 cm^{-1}, indicating the formation of an imide C-N bond.

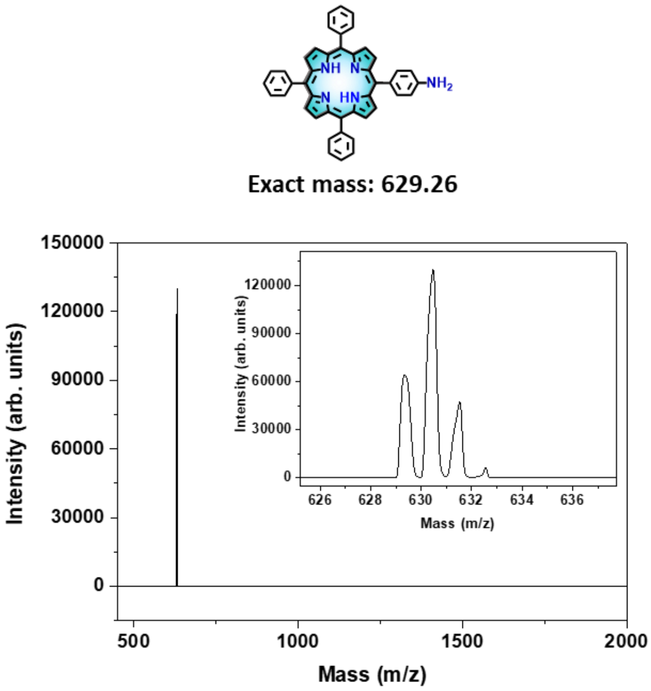

Supplementary Fig. 11. Control experiment of imide reactions without SOS surfactant micelles. The imide reactions conducted in an aqueous solution under similar experimental conditions (pH, temperature, time, and concentration) but without the use of SOS surfactant micelles. Mass spectrometry identified only the reagent molecules, indicating that the reactions did not proceed to form the imide product. This control experiment demonstrates that the confinement of porphyrin molecules on the water surface, facilitated by the micelle head groups, is crucial for enhancing their chemical reactivity.

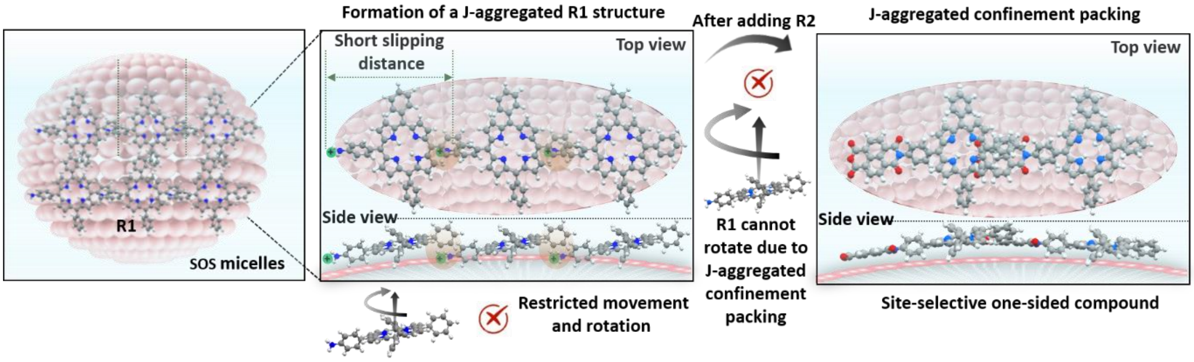

Supplementary Fig. 12. A schematic illustrating a site-selective chemical reaction on the surface of SOS micelles.

Supplementary Note 4

The selective reaction of only one anhydride group of R2 is influenced by the constrained geometry and polarized-π interactions of the J-aggregated R1 molecules on the micelle surface, which restrict the movement or rotation of the J-aggregated R1. This constrained arrangement of the porphyrin molecules facilitates the directional alignment of the R2 reagent, resulting in the selective formation of a one-sided imide product.

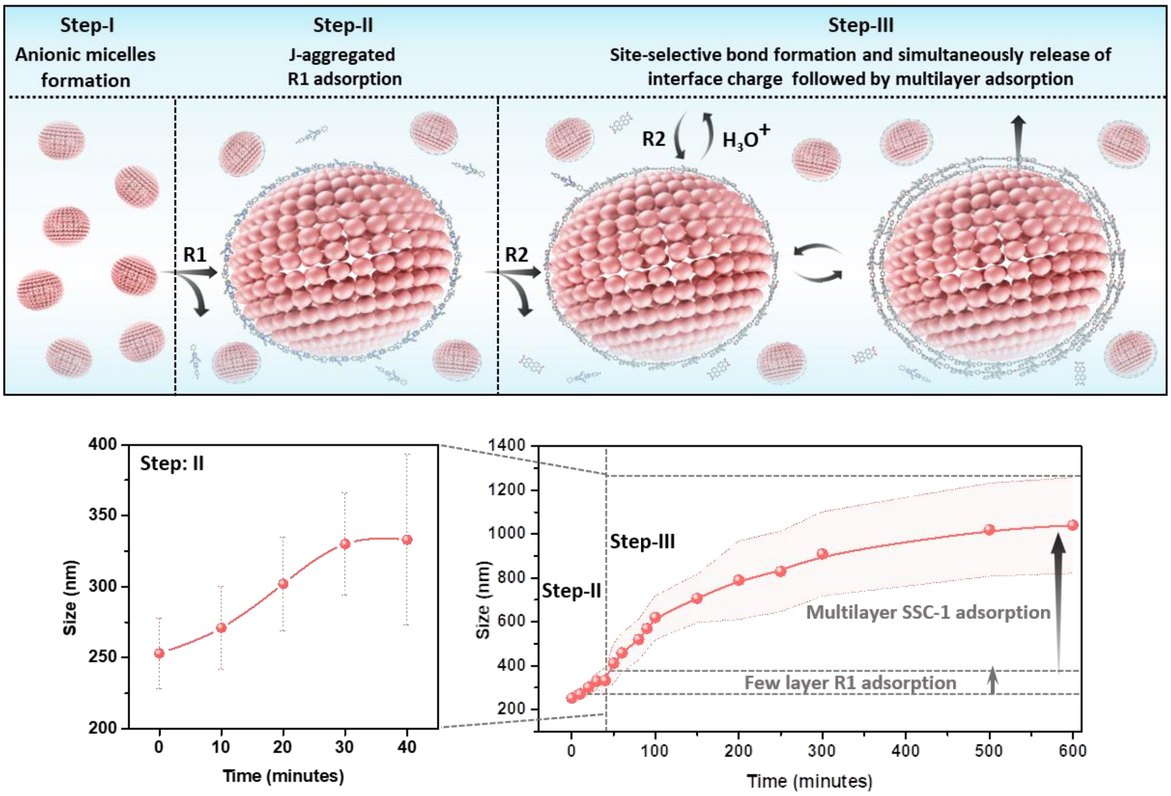

Supplementary Fig. 13. The reaction progression from Step-I to Step-III was thoroughly analyzed using in situ techniques such as Dynamic Light Scattering (DLS) and Nuclear Magnetic Resonance (NMR) spectroscopy, with an imide reaction (SSC-1) serving as a representative example of the layer-by-layer assembly mechanism.

Supplementary Note 5

Step-I: In this step, anionic micelles of SOS were formed by dissolving SOS in an aqueous solution. The ^{1}H NMR spectra showed characteristic peaks corresponding to the SOS surfactant within the micelles, providing a baseline for monitoring subsequent changes.

Step-II: Following the formation of micelles, protonated 4-(5,10,15-triphenylporphyrin-20-yl)aniline (R1) molecules were introduced into the aqueous solution at pH ~1.2. The ^{1}H NMR spectra showed a downfield shift in the characteristic peak of the SOS surfactant within 30 minutes of introducing R1, indicating increased electron density at the micelle surface due to

the adsorption of the R1 molecules. This shift is attributed to the electrostatic interaction between the negatively charged head groups (SO_4^-) of the SOS surfactant and the protonated R1 molecules. Concurrently, time-dependent DLS measurements revealed an increase in micelle size upon the introduction of R1, confirming the adsorption process. After several layers of R1 were adsorbed, no further adsorption was observed after 30 minutes, as indicated by the stabilization of the hydrodynamic diameter of the R1-SOS micelle assembly (average size 325 nm). This stability suggests that the protonated R1 molecules effectively screened the negative surface charge of the SOS micelles, leading to charge neutralization.

Step-III: The addition of naphthalene tetracarboxylic dianhydride (R2) molecules triggered a rapid increase in micelle size, as observed in time-dependent DLS measurements, which stabilized at approximately 1080 nm after 10 hours. This size increase is indicative of multilayer adsorption, driven by the chemical reaction between R1 and R2 on the micelle surface, resulting in imide bond formation and the release of H_3O^+ ions. The release of protons from the reaction sites rendered the micelle surface negatively charged, facilitating further adsorption of positively charged R1 molecules from the bulk aqueous solution.

The negative surface charge, along with the well-defined J-aggregate template structure on the micelles, served as a driving force for the continued adsorption of R1. The ongoing formation of imide bonds and subsequent charge release perpetuated the layer-by-layer growth of the multilayer site-selective product (SSC-1), which reached equilibrium after 10 hours, with a hydrodynamic diameter of approximately 1080 nm, as shown by the time-dependent DLS analysis. The observed surface charge behavior is consistent with prior studies on SOS surfactant monolayers on the water surface, including findings from ultrafast phase-sensitive interface-selective nonlinear vibrational spectroscopy, particularly sum-frequency generation(SFG) spectroscopy[3].

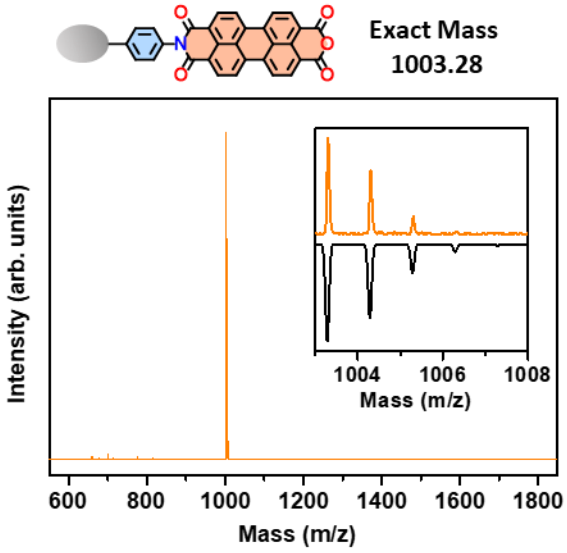

Supplementary Fig. 14. MALDI-TOF mass spectra of SSC-2 synthesized on the anionic micelles surface; inset shows HR-MALDI-TOF mass spectra.

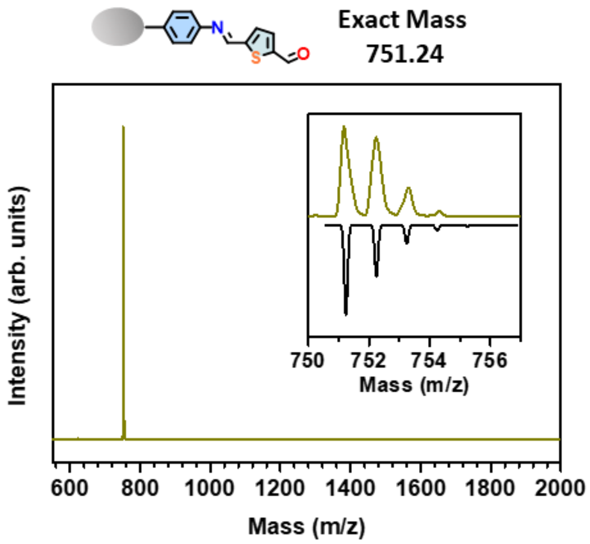

Supplementary Fig. 15. MALDI-TOF mass spectra of SSC-3 synthesized on the anionic micelles surface; inset shows HR-MALDI-TOF mass spectra.

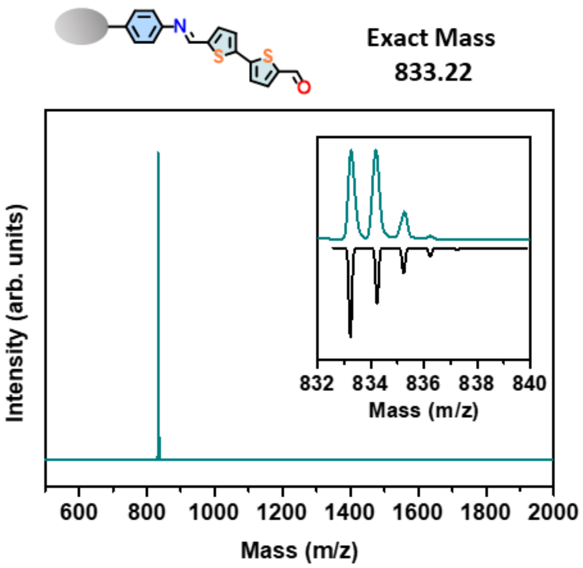

Supplementary Fig. 16. MALDI-TOF mass spectra of SSC-4 synthesized on the anionic micelles surface; inset shows HR-MALDI-TOF mass spectra.

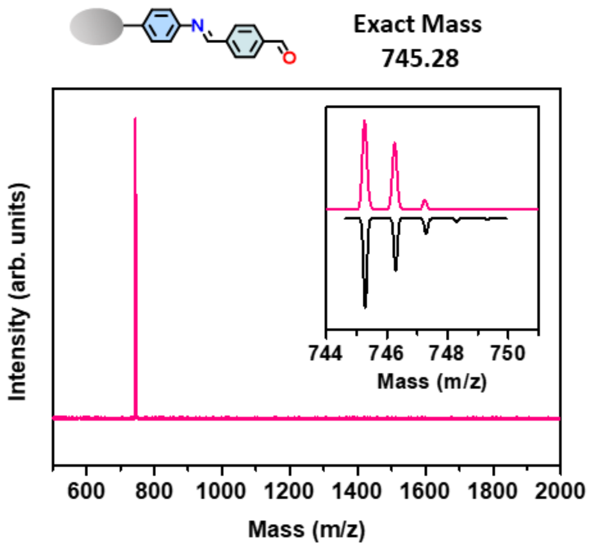

Supplementary Fig. 17. MALDI-TOF mass spectra of SSC-5 synthesized on the anionic micelles surface; inset shows HR-MALDI-TOF mass spectra.

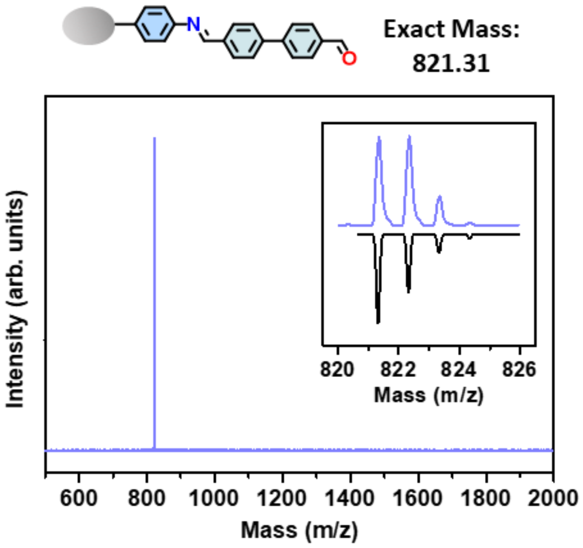

Supplementary Fig. 18. MALDI-TOF mass spectra of SSC-6 synthesized on the anionic micelles surface; inset shows HR-MALDI-TOF mass spectra.

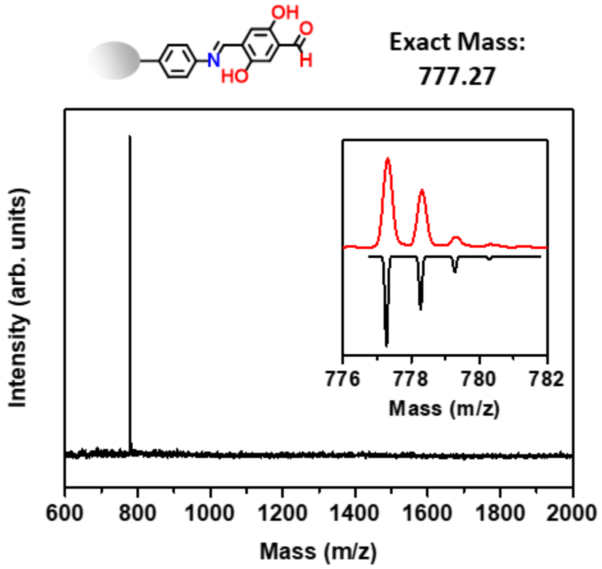

Supplementary Fig. 19. MALDI-TOF mass spectra of SSC-7 synthesized on the anionic micelles surface; inset shows HR-MALDI-TOF mass spectra.

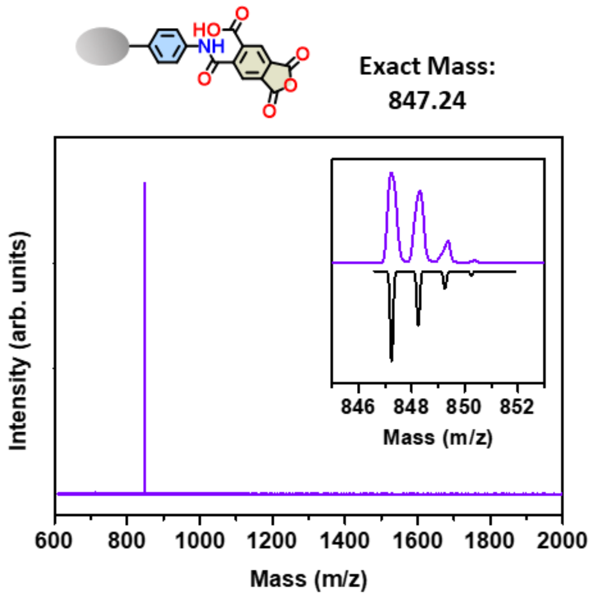

Supplementary Fig. 20. MALDI-TOF mass spectra of SSC-8 synthesized on the anionic micelles surface; inset shows HR-MALDI-TOF mass spectra.

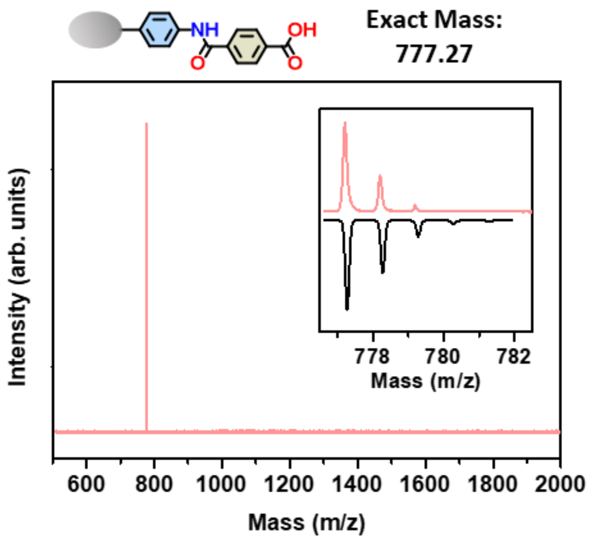

Supplementary Fig. 21. MALDI-TOF mass spectra of SSC-9 synthesized on the anionic micelles surface; inset shows HR-MALDI-TOF mass spectra.

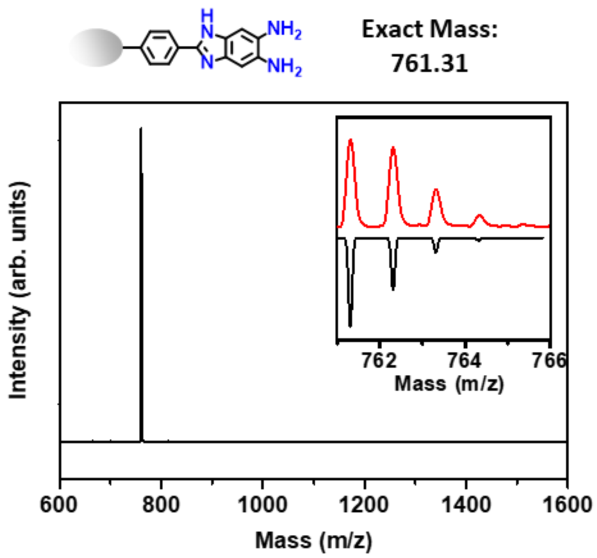

Supplementary Fig. 22. MALDI-TOF mass spectra of SSC-10 synthesized on the cationic micelles surface; inset shows HR-MALDI-TOF mass spectra.

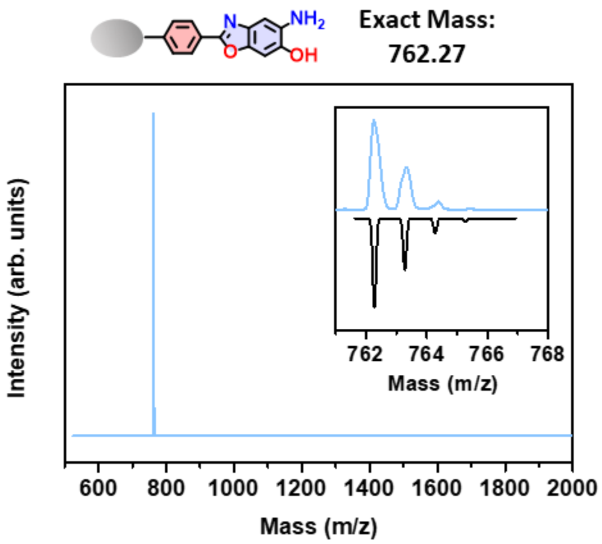

Supplementary Fig. 23. MALDI-TOF mass spectra of SSC-12 synthesized on the cationic micelles surface; inset shows HR-MALDI-TOF mass spectra.

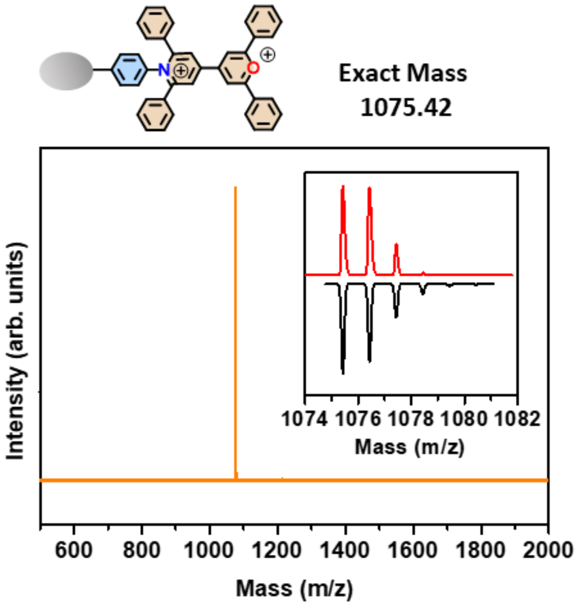

Supplementary Fig. 24. MALDI-TOF mass spectra of SSC-13 synthesized on the anionic micelles surface; inset shows HR-MALDI-TOF mass spectra.

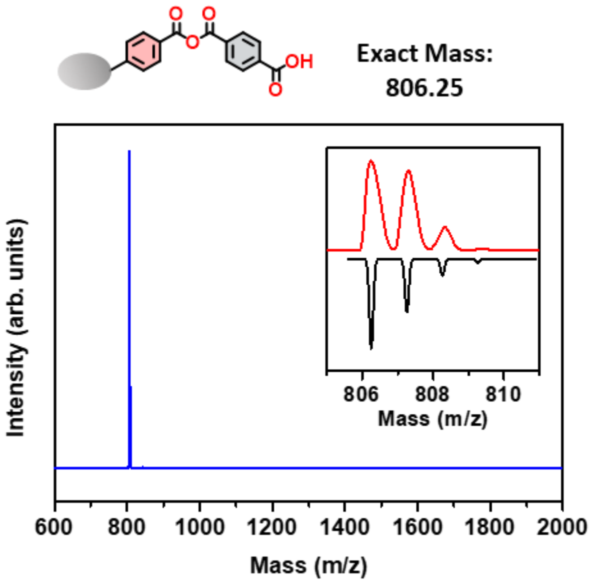

Supplementary Fig. 25. MALDI-TOF mass spectra of SSC-14 synthesized on the cationic micelles surface; inset shows HR-MALDI-TOF mass spectra.

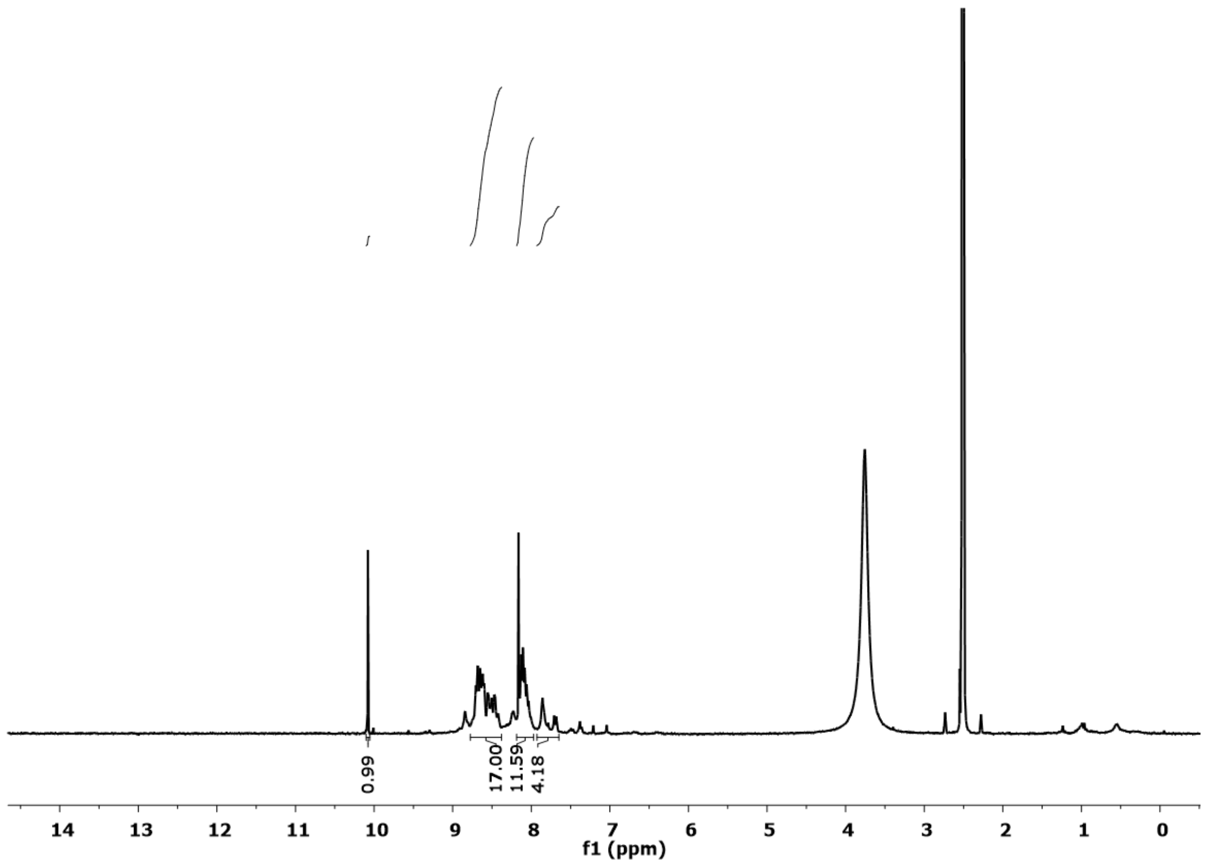

Supplementary Fig. 26. ^{1}H NMR spectra (300 MHz) of SSC-3 in DMSO-d^{6} solvent.

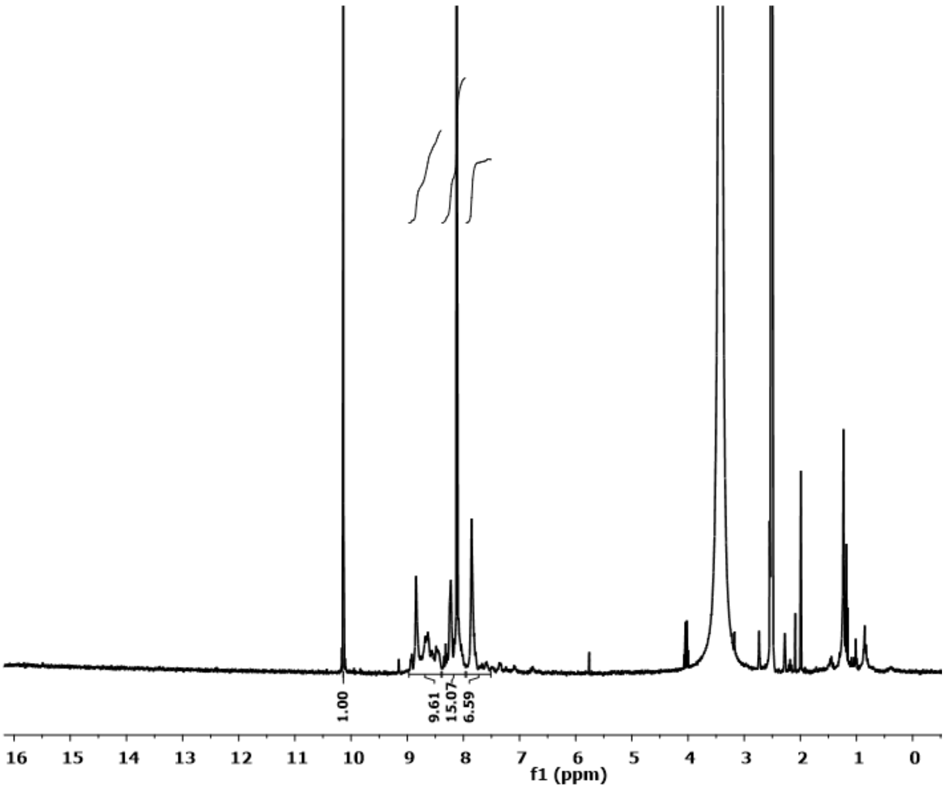

Supplementary Fig. 27. ^{1}H NMR spectra (300 MHz) of SSC-5 in DMSO-d^{6} solvent.

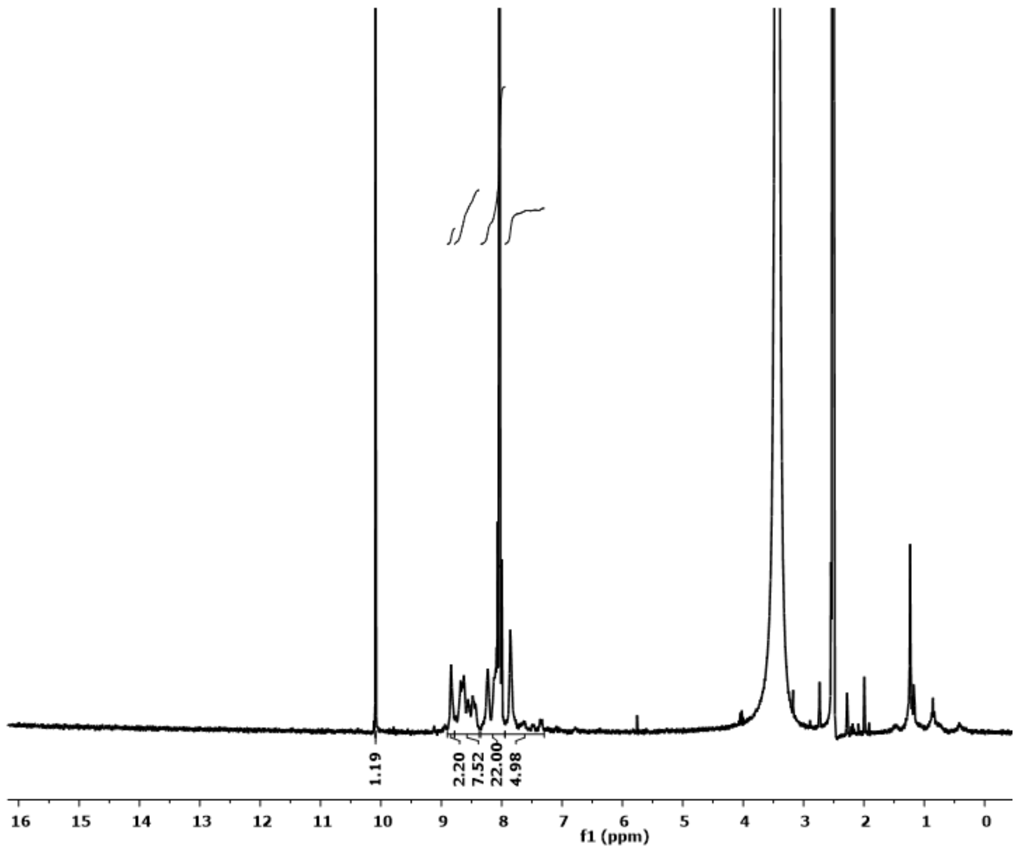

Supplementary Fig. 28. ^{1}H NMR spectra (300 MHz) of SSC-6 in DMSO-d^6 solvent.

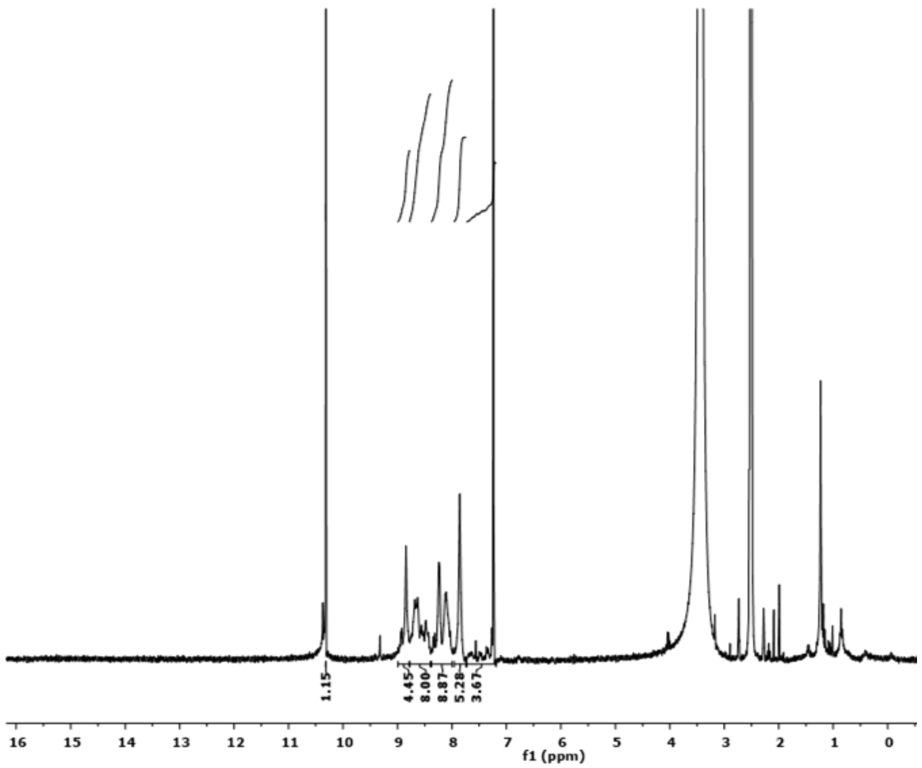

Supplementary Fig. 29. ^{1}H NMR spectra (300 MHz) of SSC-7 in DMSO-d^6 solvent.

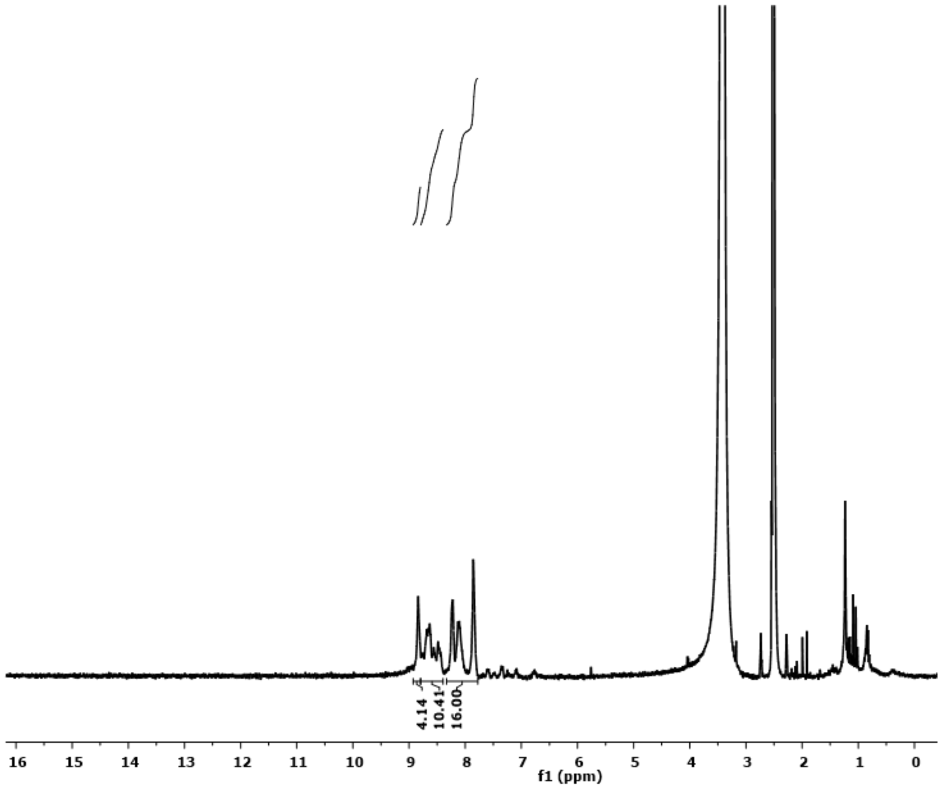

Supplementary Fig. 30.^{1}H NMR spectra (300 MHz) of SSC-8 in DMSO-d^{6} solvent.

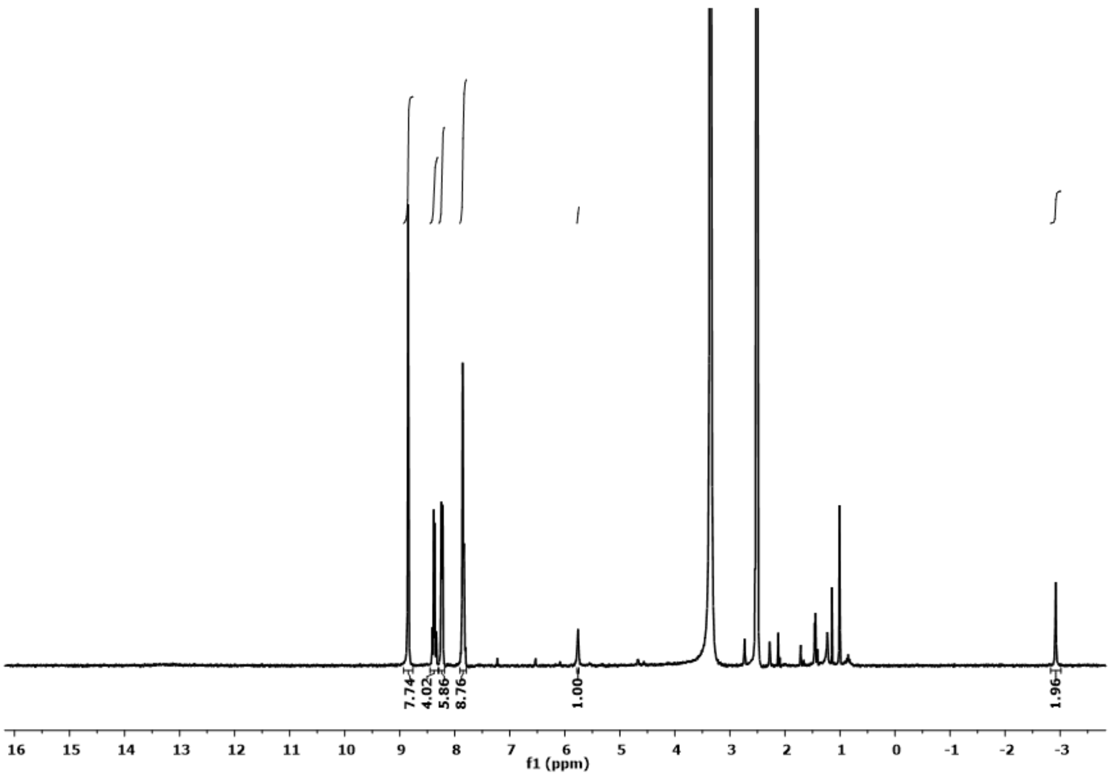

Supplementary Fig. 31. ^{1}H NMR spectra (300 MHz) of SSC-10 in DMSO-d^6 solvent.

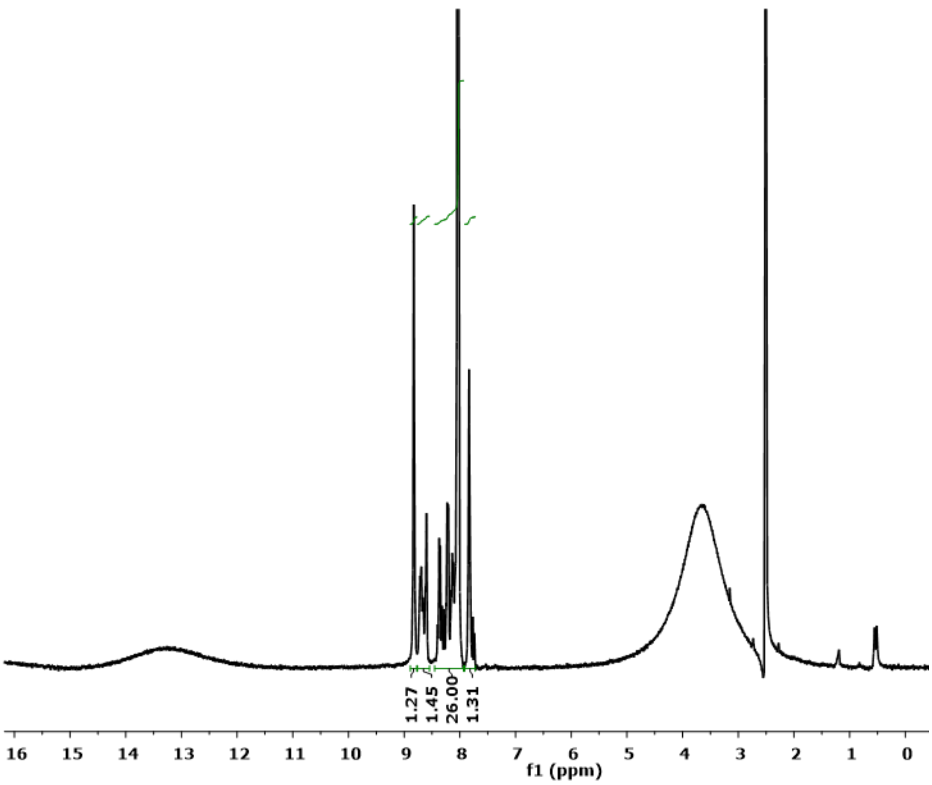

Supplementary Fig. 32. ^{1}H NMR spectra (300 MHz) of SSC-12 in DMSO-d^6 solvent.

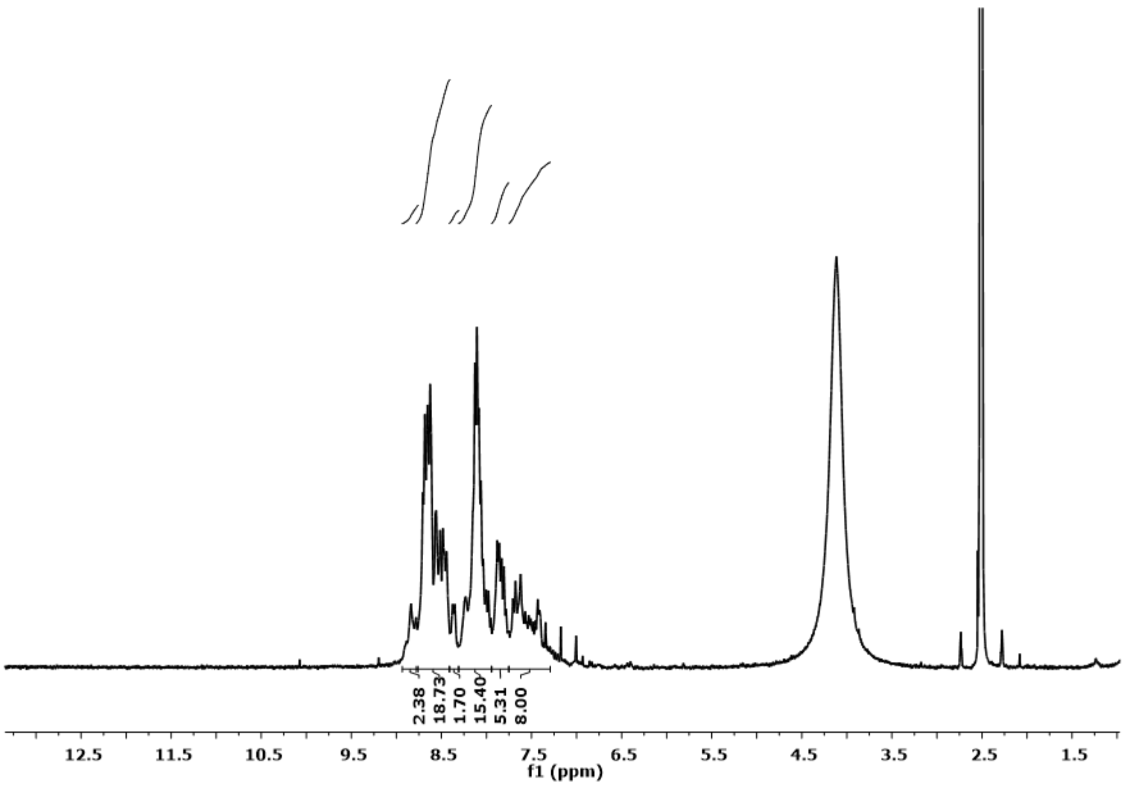

Supplementary Fig. 33. ^{1}H NMR spectra (300 MHz) of SSC-13 in DMSO-d^6 solvent.

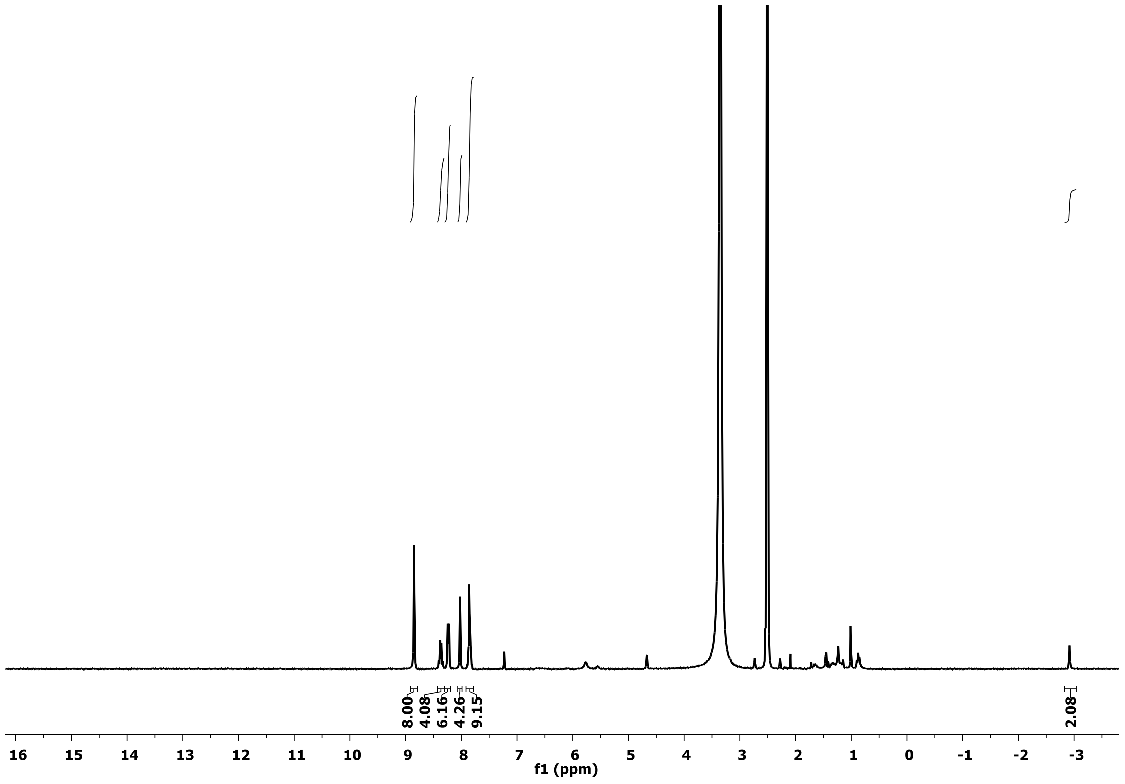

Supplementary Fig. 34. ^{1}H NMR spectra (300 MHz) of SSC-14 in DMSO-d^{6} solvent.

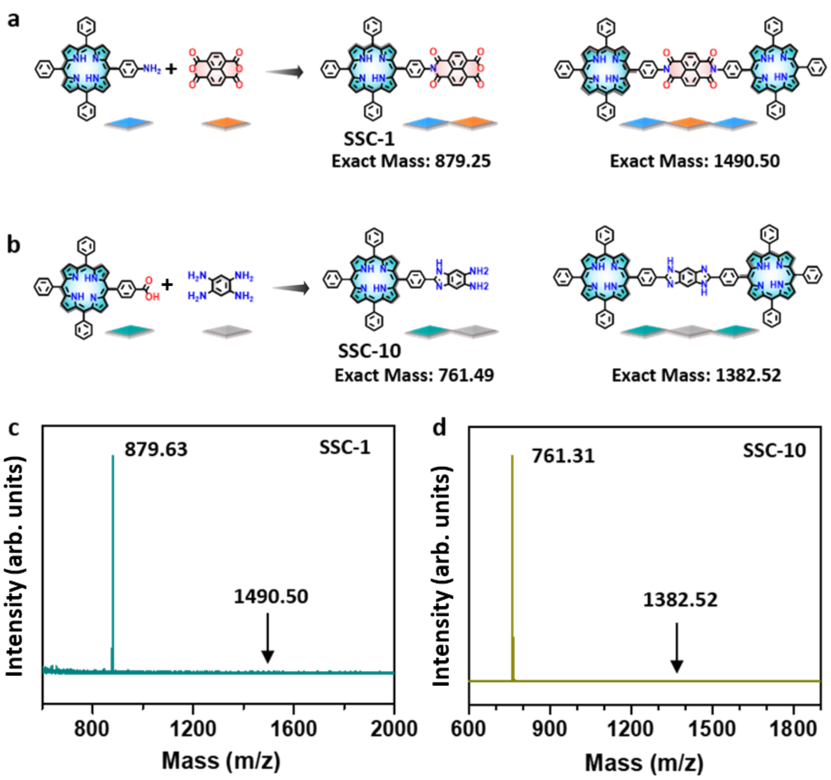

Supplementary Fig. 35. A schematic illustrating the site-selective chemical reaction for scale-up of (a) SSC-1 and (b) SSC-10 on the water surface, including the expected two-sided product. (c) HR-MALDI-TOF mass spectra of the site-selective products SSC-1 and SSC-10.

Supplementary Note 6

For the anionic SOS surfactant micelles, we scaled up the synthesis of SSC-1, achieving an isolated yield of 93% (6.5 mg). Similarly, for the cationic CTAB surfactant micelles, we scaled up the synthesis of SSC-10, obtaining an isolated yield of 94% (5.4 mg). Here, we would like to mention that the solubility of the reactants, 4-(5,10,15-triphenylporphyrin-20-yl)aniline (R1) and 5-(4-carboxyphenyl)-10,15,20-(triphenyl) porphyrin (R3), is low in water, which limited the scale-up of the reactions and resulted in these product quantities. The selectivity in the scaled-up reactions (SSC-1 and SSC-10) was maintained at over 99%, as confirmed by MALDI-TOF MS and NMR spectroscopy, with no evidence of two-sided imide or imidazole products. Only the site-selective products, SSC-1 and SSC-10, were observed.

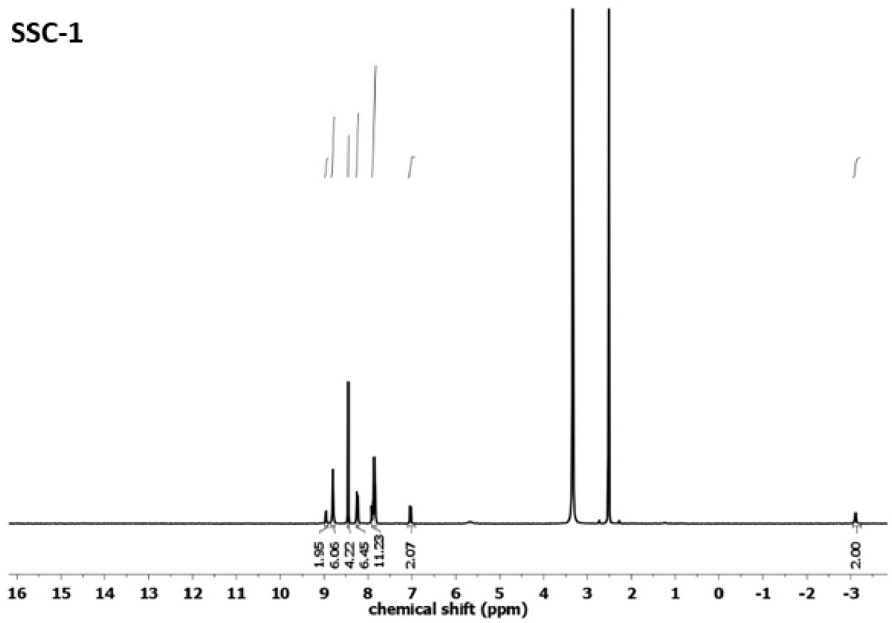

Supplementary Fig. 36 ^{1}H NMR spectra (300 MHz) of SSC-1 in DMSO-d^6 solvent.

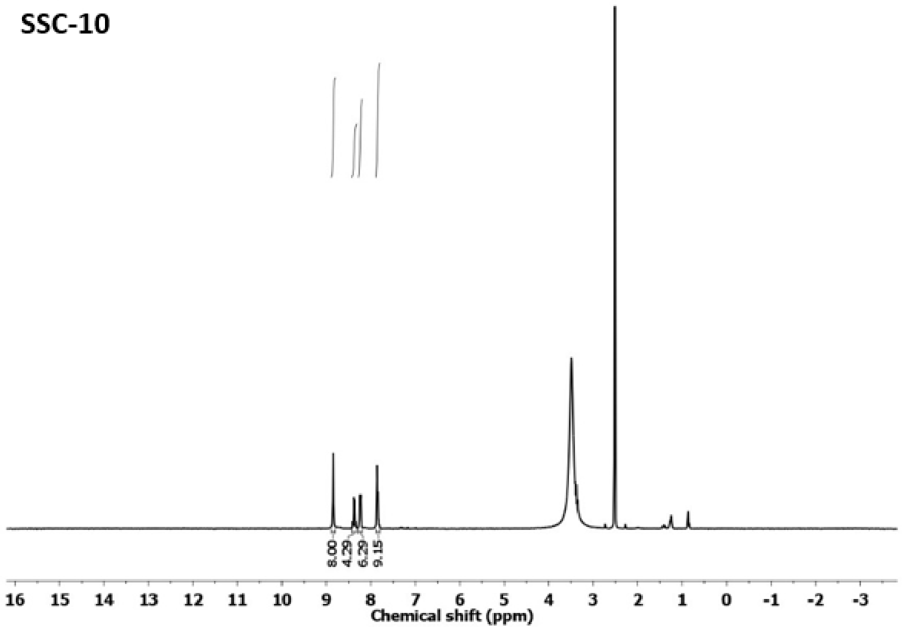

Supplementary Fig. 37. ^{1}H NMR spectra (300 MHz) of SSC-10 in DMSO-d^6 solvent.

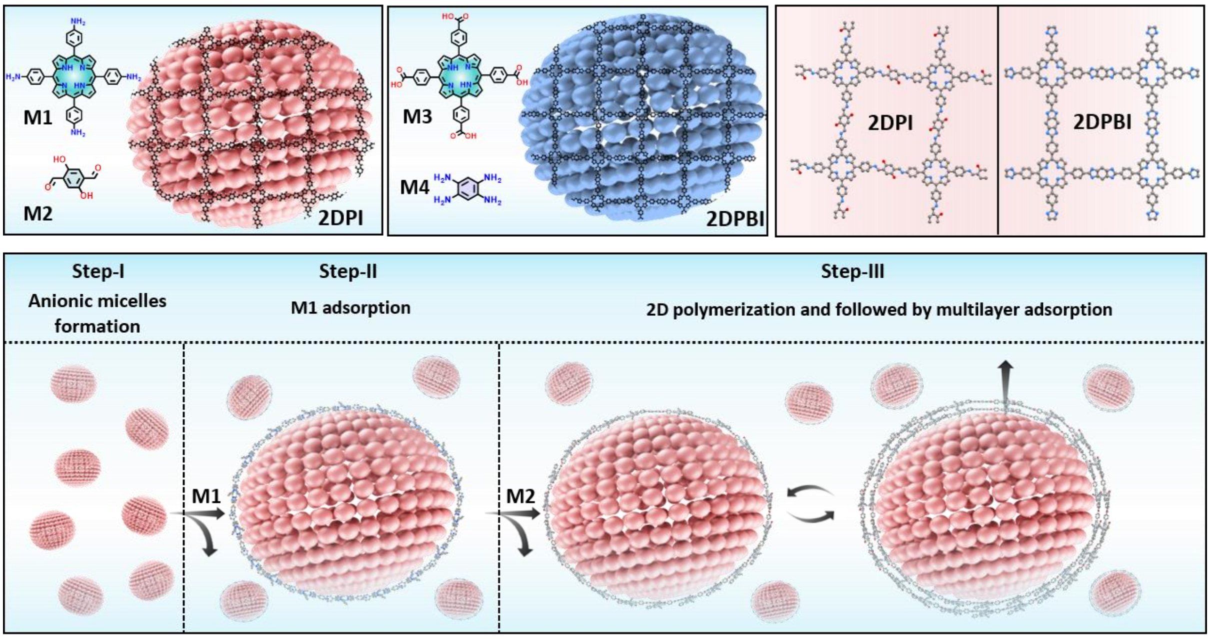

Supplementary Fig. 38. Dynamic micellar interface induced by surface charge to favor multilayer 2D polymer growth. The schematic illustrates the stepwise process of 2D polymerization and multilayer 2D polymer (2DPI and 2DPBI) growth on cationic and anionic micelles. The process involves three key steps.

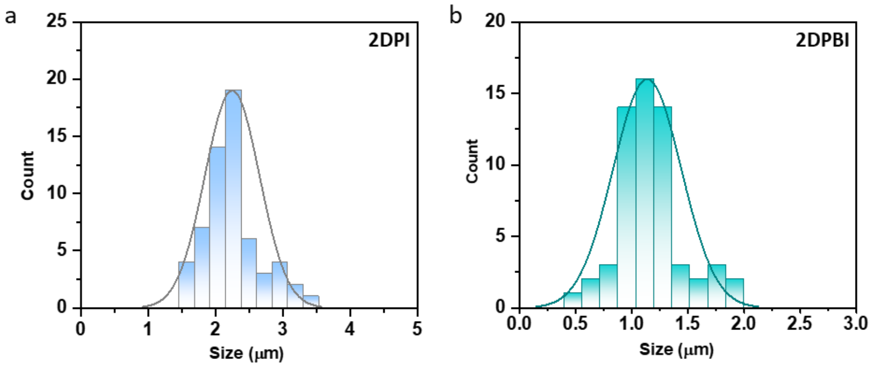

Supplementary Fig. 39. Size distribution histograms of 2DPI (left) and 2DPBI (right). The average size of 2DPI is approximately 2.25 μm with a standard deviation of 0.40 μm, while the average size of 2DPBI is about 1.14 μm with a standard deviation of 0.30 μm.

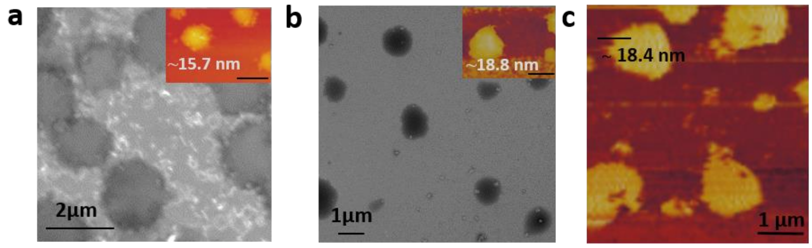

Supplementary Fig. 40. Field-emission scanning electron microscopy (FE-SEM) images of (a) 2DPI and (b) 2DPBI reveal circular, sheet-like morphologies with uniform sizes of approximately 2 µm and 1 µm, respectively (the darker areas represent the 2DPI and 2DPBI circular sheets). Atomic Force Microscopy (AFM) measurements show a thickness of about 15.7 nm for 2DPI (scale bar: 2 µm) and about 18.8 nm for 2DPBI (scale bar: 0.5 µm) and (c) zoomed AFM images of 2DPBI, indicating the formation of layer-stacked 2D polymers.

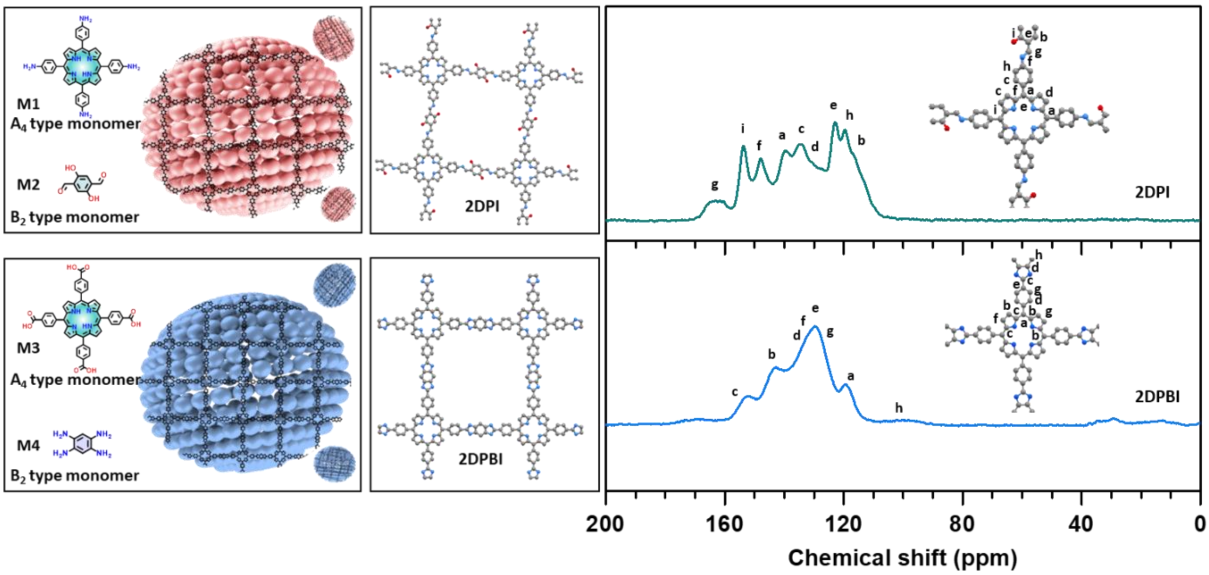

Supplementary Fig. 41. Schematic representations of the synthesis of 2DPI on anionic micelles and 2DPBI on cationic micelles, along with the solid-state ^{13}C NMR spectra of 2DPI and 2DPBI (The lines were assigned to the atomic positions using ACD/Labs software (Advanced Chemistry Development Inc., 2017)).

Supplementary Note 7

We scaled up 2D polymerization reactions (2DPI and 2DPBI) using both cationic and anionic surfactant micelles to further demonstrate the versatility of the approach. For 2D polymerization, we used porphyrin monomers 5,10,15,20-(tetra-4-aminophenyl)porphyrin (M1) and 5,10,15,20-(tetra-4-carboxyphenyl)porphyrin (M3). The isolated yields for the 2D polymers, 2DPI and 2DPBI, were 24.9 mg and 31.2 mg (92% and 94% yield), respectively. Solid-state ^{13}C NMR spectroscopy confirmed the presence of imine and imidazole linkages, verifying the linkage specificity of the polymerized products. These results indicate that while solubility limits the scale of some reactions, the methodology maintains high selectivity and efficiency.

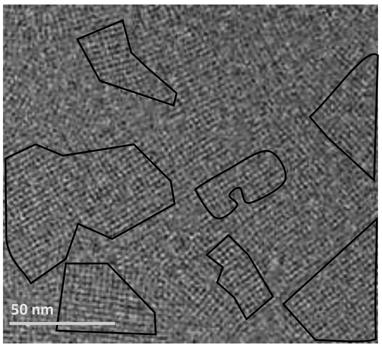

Supplementary Fig. 42. TEM image showing different alignments of crystal domains in the 2D polymer (2DPI). The observed variations in domain orientation are influenced by the angular curvature of the micelle template, which can reduce crystallinity and lead to a polycrystalline nature with domains aligned in multiple directions.

Supplementary Note 8

The curvature of the micelle surface, particularly angular curvature, significantly influences the packing and arrangement of porphyrin molecules. Larger micelles have lower curvature, providing a more planar surface, which allows for a more even distribution with less distortion, better organization of the porphyrin self-assembled structure, and stable adsorption of porphyrins. This structured assembly is essential for developing 2D polymers with larger domain sizes and higher crystallinity. In our study, larger micelles, such as those formed by the M1-SOS anionic surfactant (~310 nm), facilitate the pre-assembly of monomers over more extensive domain areas, promoting higher crystallinity in 2DPI. In contrast, smaller micelles, such as those formed by the M3-CTAB cationic surfactant (~106 nm), are constrained by their limited surface area and increased angular curvature, leading to reduced crystallinity and a tendency towards polycrystallinity with smaller circular-sheet domains in 2DPBI.

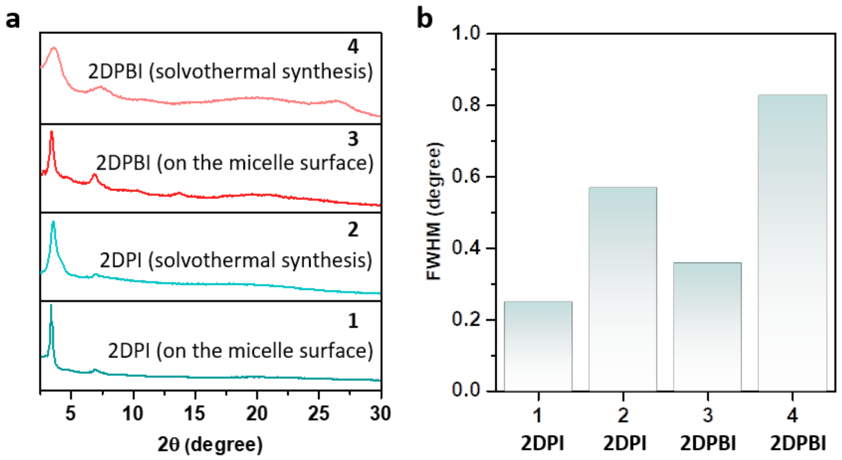

Supplementary Fig. 43. (a) PXRD patterns comparing the crystallinity of 2D polymers synthesized by micelle-assisted and solvothermal methods. The sharper peaks observed in the micelle-assisted synthesis indicate higher crystallinity. (b) Comparison of FWHM values for the (100) plane reflections, showing lower FWHM for micelle-assisted synthesis (bars 1 and 3) and higher FWHM for solvothermal synthesis (bars 2 and 4).

Supplementary Note 9

A comparative analysis was performed to compare the crystallinity of 2D polymers using powder X-ray diffraction (PXRD) and transmission electron microscopy (TEM). Specifically, 2DPI and 2DPBI were synthesized using both a micelle-assisted approach and the conventional solvothermal bulk organic synthesis commonly reported in the literature. PXRD analysis revealed that the Full Width at Half Maximum (FWHM) values for the (100) plane of the micelle-assisted synthesis of 2DPI and 2DPBI were 0.25° and 0.36°, respectively. In contrast, the solvothermal synthesis approach showed broader peaks with FWHM values of 0.57° for 2DPI and 0.83° for 2DPBI, indicating lower crystallinity, as higher FWHM values correspond to smaller crystallite sizes and less ordered structures.

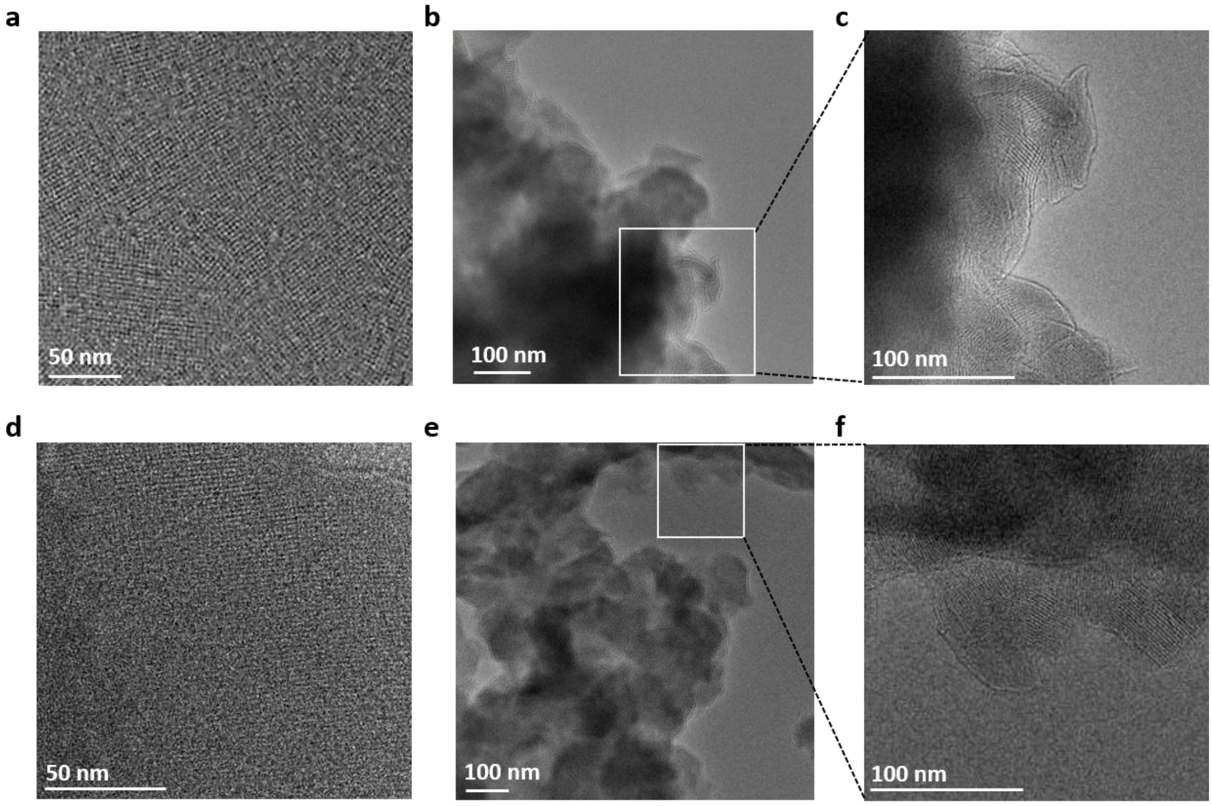

Supplementary Fig. 44. (a) TEM images comparing the crystallinity of 2D polymers synthesized by micelle-assisted and solvothermal methods. Images (a) and (d) represent 2DPI and 2DPBI synthesized via the micelle-assisted approach, showing well-defined, continuous lattice fringes over extended areas. In contrast, images (b), (c), (e), and (f) show 2DPI and 2DPBI synthesized via the solvothermal approach, exhibiting less distinct lattice patterns with noticeable disorder, indicative of smaller crystalline domains and lower overall crystallinity.

Supplementary Note 10

Additionally, TEM imaging showed that the micelle-assisted synthesis of 2DPI and 2DPBI exhibited well-defined and continuous lattice fringes over extended areas (Figure R2a and R2d), characteristic of high crystallinity and long-range order. In contrast, the 2DPs (2DPI and 2DPBI) synthesized via the solvothermal approach displayed less distinct lattice patterns with noticeable disorders (Figure R2b-c and R2d-e), indicative of smaller crystalline domains and lower overall crystallinity.

Supplementary References

1. Kandambeth, S. *et al.* Enhancement of chemical stability and crystallinity in porphyrin-containing covalent organic frameworks by intramolecular hydrogen bonds. *Angew. Chem., Int. Ed.*, 13290-13294 (2013).

2. Ranjeesh, K. C. *et al.* Imidazole-linked crystalline two-dimensional polymer with ultrahigh proton-conductivity. *J. Am. Chem. Soc.* **141**, 14950-14954 (2019).

3. Prasoon, A. *et al.* Site-selective chemical reactions by on-water surface sequential assembly. *Nat. Commun.* **14**, 8313 (2023).
